# The Mclust Analysis of Tumor Budding Unveils the Role of the Collagen Family in Cervical Cancer Progression

**DOI:** 10.3390/life14081004

**Published:** 2024-08-13

**Authors:** Olive EM Lee, Tan Minh Le, Gun Oh Chong, Junghwan Joshua Cho, Nora Jee-Young Park

**Affiliations:** 1 Department of Biomedical Science, Graduate School, Kyungpook National University, Daegu 41944, Republic of Korea; 2 BK21 Four Program, School of Medicine, Kyungpook National University, Daegu 41944, Republic of Korea; 3 Clinical Omics Institute, Kyungpook National University, Daegu 41405, Republic of Korea; 4 Department of Obstetrics and Gynecology, School of Medicine, Kyungpook National University, Daegu 41944, Republic of Korea; 5 Department of Obstetrics and Gynecology, Kyungpook National University Chilgok Hospital, Daegu 41404, Republic of Korea; 6 Department of Pathology, School of Medicine, Kyungpook National University, Daegu 41944, Republic of Korea; 7 Department of Pathology, Kyungpook National University Chilgok Hospital, Daegu 41404, Republic of Korea

**Keywords:** cervical cancer, Mclust, tumor budding, collagen type VI, EMT, ECM

## Abstract

In RNA-seq data analysis, condensing the gene count matrix size is pivotal for downstream investigations, particularly pathway analysis. For this purpose, harnessing machine learning attracts increasing interest, while conventional methodologies depend on *p*-value comparisons. In this study, 20 tissue samples from real-world cervical cancers were subjected to sequencing, followed by the application of the Mclust algorithm to delineate an optimal cluster. By stratifying tumor budding into high and low groups and quantifying the epithelial-to-mesenchymal transition (EMT) score to scrutinize tumor budding, we discerned 24 EMT-related genes, with 5 showing strong associations with cervical cancer prognosis. Our observations elucidate a biological flow wherein EMT, Matrix Metallopep-tidase 2 (MMP2), and extracellular matrix (ECM) degradation are interconnected, ultimately leading to collagen type VI and exacerbating the prognosis of cervical cancer. The present study underscores an alternative method for selecting useful EMT-related genes by employing an appropriate clustering algorithm, thereby avoiding classical methods while unveiling novel insights into cervical cancer etiology and prognosis. Moreover, when comparing high and low tumor budding, collagen type VI emerges as a potential gene marker for the prognosis of cervical cancer.

## 1. Introduction

Cervical cancer still poses a significant global burden, with incidence and mortality remaining substantially above the thresholds outlined by the WHO initiative for cervical cancer elimination in most countries [1]. This ongoing disparity underscores the imperative for sustained research efforts and targeted interventions.

In cancer research, RNA-seq stands out as the pre-eminent method for comprehensive genome-wide gene expression analysis [2], representing a powerful tool for probing gene transcripts across diverse applications [3]. While RNA-seq provides valuable insights into the molecular intricacies of cervical cancer, the vast gene count matrix it generates necessitates strategic gene selection. However, conventional clustering approaches in high-dimensional RNA-seq data often yield inconclusive results due to the curse of dimensionality [4,5]. 

Therefore, clustering techniques have been widely adopted for feature selection in cancer research to address this challenge [4,6,7]. A study [8] utilized breast cancer data to predict recurrent events by applying SOMs (Self-Organizing Maps), k-means, and a cluster network. Another investigation [9] employed hierarchical clustering to classify disease groups in cervical cancer based on genotype frequencies. While numerous clustering algorithms are available, the Mclust algorithm [10,11] can be applied in diverse research areas, including the grouping of rectal cancers [12], identification of immune subtypes from Pan-Cancer gene expression data [13], comparison of clusters in renal cell carcinoma [14], selection of the top 25 species in baseline samples [15], and assessment of the likelihood of different Gaussian mixtures in cervical cancer [16]. 

The term “tumor budding” (TB) is the presence of single cells or small clusters of cells at the leading edge of the tumor [17]. High TB (HTB) is a notable risk factor for nodal involvement and unfavorable outcomes, often indicating epithelial–mesenchymal transition (EMT) [18,19]. Numerous studies across various cancer types have examined this phenomenon to elucidate its prognostic significance [20,21,22].

Additionally, there is mounting evidence from studies suggesting that collagen within tumors plays a significant role in cancer development and progression [23]. For instance, a recent study [24] has indicated that tumor-derived type III collagen is essential for restoring tumor cell proliferation by modifying the ECM. Further investigations yielded various findings [25,26]. Despite these advancements, the specific relationship between the collagen family and TB in cervical cancer remains unexplored. 

In our current study which builds on previous research [27,28], our objectives encompassed two pivotal factors. First, instead of analyzing the entire matrix, we focused on an optimal cluster to unveil significant insights within our real-world clinical data. Second, HTB might promote the spread of cancer cells to lymph nodes, increasing the risk of a negative prognosis. Our study aimed to elucidate these inquiries by computing an EMT score [29,30], drawing inspiration from previous studies [17,19] given the close association between TB and EMT. We concentrated on an optimal cluster by employing the Mclust unsupervised algorithm, thereby revealing crucial insights into our clinical data between HTB and low TB (LTB). Subsequently, pathway analysis unveiled the presence of collagen type VI in TB induction.

## 2. Materials and Methods

Figure S1 shows the entire process used for the present study. 

### 2.1. Sample Acquisition

This study received approval from the Institutional Review Board of Kyungpook National University Chilgok Hospital (protocol code KNUMC 2020-10-003 and 19-OCT-2020 of approval). It spanned from February 2011 to September 2021, involving the collection of 20 cervical cancer tissue samples immediately after radical hysterectomy with pelvic and/or paraaortic lymphadenectomy to treat early-stage and locally advanced cervical cancer. The exclusion criteria comprised patients with a history of preoperative chemotherapy, radiotherapy, or synchronous malignancies. The primary clinical parameters itemized herein include key factors such as age, FIGO stage, histology, TB, cancer stage, lymph-vascular space invasion (LVSI), and recurrence-free survival (RFS).

### 2.2. RNA Extraction

RNA was extracted from 5 µm sections of each sample using the ReliaPrep FFPE RNA Miniprep System (Promega, Madison, WI, USA) following the manufacturer’s instructions. Libraries were generated using the TruSeq RNA Exome Kit(Illumina, San Diego, CA, USA) and RNA sequencing was performed on the NovaSeq 6000 system (Illumina, San Diego, CA, USA). RNA quality and quantity were assessed using the Qubit 4 Fluorometer (Thermo Fisher Scientific, Waltham, MA, USA) and the Agilent Bioanalyzer (Agilent Technologies, Santa Clara, CA, USA).

### 2.3. Alignment and Quality Control

Paired-end raw reads from NovaSeq 6000 were processed by removing adapter sequences and low-quality reads (Phred score < 20) and trimming reads <50 bp using Cutadapt (v.2.8). Clean reads were aligned to the human genome (UCSChg38) with STAR (v.2.7.10a), and gene counts were obtained using HTseq-count (v.2.0.1).

### 2.4. Preprocessing the Gene Count Matrix 

We implemented a filtering step to exclude genes with a cumulative count of <1. The R package DESeq2 (version number 1.44.0) [31] was employed for the identification of differentially expressed genes (DEGs), using TB as the reference for comparing groups of HTB and low TB (LTB). The LTB group consisted of 6 patients, while the HTB group comprised 14 patients, defined as having <3 buds and ≥4 buds, respectively.

A quantification gene matrix was derived from samples of cervical cancer patients, encompassing 20 individuals. Gene symbols were matched with Ensembl gene IDs [32] using the R package AnnotationDbi [33]. In cases of duplicated gene symbols, they were substituted with the mean value to ensure unique identification. A compilation of immune-related genes was obtained from the Innate Immune DB in December 2023 [34], providing 4677 unique gene symbols. 

### 2.5. Calculating EMT Score and Clustering Algorithms

We adapted the algorithm proposed by Bozorgui et al. [30] to compute the EMT score for each gene within the list of 76 EMT-related genes. Additionally, we employed eight clustering algorithms—Mclust [10,11], hierarchical clustering with the Complete-linkage and the Ward.D method, k-means, DBSCAN [35], KNN-based Louvain community detection [36], hierarchical k-means [37], and fuzzy analysis (FANNY) clustering [38]—in R to compare cluster labels and EMT scores across algorithms. The Mclust algorithm, implemented in R [11], determined the number of clusters (k) using the Bayesian Information Criterion (BIC) [10,39,40]. By leveraging the ‘uncertainty’ metric provided by the Mclust package, we identified the optimal cluster for further analysis.

### 2.6. Pathway Analysis and Validation

We utilized R for statistical analysis, such as the Rank-Sum Test, PERMANOVA, and the linear regression model. For further pathway analysis, we utilized Enrichr [41], MSigDB [42], STRING [43], g:Profiler [44], ShineyGo [45], Pathway Commons [46], the GIPIA2 platform [47], and the Kaplan-Meier plotter [48]. Furthermore, the aforementioned seven additional clustering algorithms were used to confirm if they also lead to the identification of the collagen family pathway or COL6A2′s involvement.

## 3. Results

### 3.1. Clinical Data

The dataset comprised 20 samples in total. Of these, 6 samples corresponded to LTB, while the remaining 14 samples were categorized as HTB. Table S1 provides more comprehensive details regarding our clinical data.

### 3.2. Gene Count Matrix and Symbol

The initial matrix of gene counts comprised 64,252 genes across 20 samples. Subsequently, genes with zero counts across all samples were removed. We utilized the R package AnnotationDbi [33] to obtain gene symbols. We retained unique symbols to address duplicate gene symbols, replacing the counts of repeated symbols with their mean value. Consequently, our final list of unique gene symbols encompassed 15,497 genes, forming a 15,497 × 20 matrix. 

### 3.3. Running Deseq2

DESeq2 was utilized to assign samples to specific groups within our clinical dataset, namely LTB and HTB. This package provided informative metrics, including baseMean, log2FoldChange, lfcSE, stat, *p*-value, and adjusted *p*-value (padj).

### 3.4. Labeling as Immune-Specific Genes

We acquired the list of immune genes from the Innate Immune DB [34], comprising 4677 unique gene symbols. Out of these, 3079 genes overlapped with our gene count matrix. Consequently, our gene count matrix of 15,497 genes includes 3079 immune genes, distinguished by a specific variable label.

### 3.5. Choice of Number of Clusters (k)

The Mclust() function, a part of the Mclust package, evaluates 14 models detailed in Figure S2 to determine the one that best fits the data. Although various model selection methods such as the Akaike Information Criterion (AIC) and likelihood ratio can be utilized, the Bayesian Information Criterion (BIC) is effective in model-based clustering [39,49]. The Mclust() function implements the BIC to identify the optimal model as defined by Equation (1) [50]:

(1) $$BIC=-2\log\left(L\right)+mlog\left(n\right),$$

where log(*L*) represents the maximized log-likelihood for the model and dataset, *m* denotes the number of free parameters estimated within the model, and *n* represents the total number of observations in the dataset. This penalizes overly complex models with a high number of clusters.

**Figure 1:**
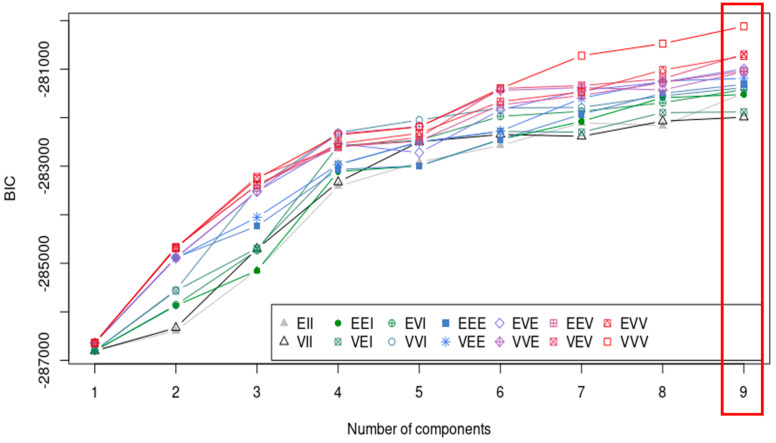
Identifying the optimal Gaussian Mixture Model (GMM) and determining the number of clusters, with k = 9 recommended, highlighted in a red box.

In the context of the BIC, a smaller BIC indicates a better-fitting model relative to its complexity. The model has the best balance between goodness of fit and model complexity. Thus, technically, the model that strikes the best balance between complexity and fit to the data is selected. By default, the function determines the optimal number of clusters (k) without requiring a specific initial k value. In our dataset, k = 9 was identified as optimal, as depicted by the red box in Figure 1. The BIC value corresponding to k = 9 was calculated as −280,255.3. Subsequently, we reanalyzed the data with k = 11, yielding a BIC value of −280,020.1. We ultimately selected k = 9 for our analysis as a lower BIC value indicates a better fit. 

### 3.6. Calculating the EMT Score and Selection of Mclust

We used the methodology outlined by [30], with additional insights from [29], to compute the EMT score for each gene within our dataset. We applied the Mclust algorithm with the optimal number of clusters (k = 9) determined previously. Additionally, seven other clustering algorithms—hierarchical clustering with the Complete-linkage and the Ward.D method, k-means, DBSCAN, KNN-based Louvain community detection, hierarchical k-means, and FANNY clustering—were used with k = 9 to compare the results with Mclust. Comprehensive details regarding this validation are provided in Table S2. In summary, we employed PERMANOVA with 1000 permutations in R, the lm test, and the Rank-Sum Test to calculate *p*-values between the EMT score and cluster labels for each clustering algorithm. The *p*-values from the Rank-Sum Test were as follows: 3.10 × 10^−6^ for Mclust, 0.05618 for hierarchical clustering with the Complete-linkage method, 0.00863 for hierarchical clustering with the Ward.D method, 0.00983 for k-means, 0.1874 for DBSCAN, 0.09424 for KNN-based Louvain, 0.00983 for hierarchical k-means, and 0.19934 for FANNY clustering. Consequently, we proceeded with our analysis using the Mclust algorithm. 

For the Mclust results, the *p*-value of 3.10 × 10^−6^ indicated a significant association between the Mclust clustering method and the EMT score. Furthermore, the *p*-value from the linear model test (lm) was 1.405 × 10^−6^, indicating consistency. These additional steps helped validate the robustness of our findings (Table S2). 

### 3.7. Application of the Mclust with the Optimal k (k = 9) and Selection of the Optimal Cluster

**Figure 2:**
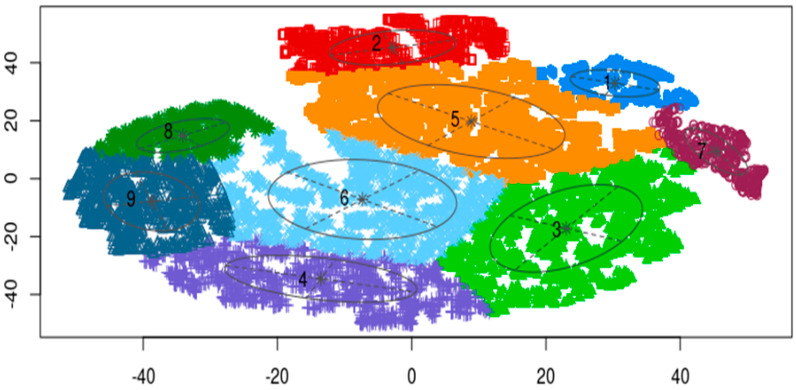
Mclust result. Cluster 1: 619 members. Cluster 2: 1133 members. Clusters 3–9: 2858, 1952, 3155, 2798, 602, 904, and 1476 members, respectively.

Figure 2 depicts the clustering output. Cluster 1 comprised 619 members, while Cluster 2 encompassed 1133 members. Clusters 3–9 consisted of 2858, 1952, 3155, 2798, 602, 904, and 1476 members, respectively. 

Subsequently, we proceeded to identify the optimal cluster for further investigation. Specifically, we utilized the “uncertainty” metric provided by Mclust(), which represents the probability of a data point being assigned to two clusters. Data points residing close to cluster boundaries may result in dual assignments, highlighting the significance of minimizing “uncertainty” to ensure accurate cluster selection. Table 1 elucidates the uncertainty values exceeding 50%. Our analysis revealed that Cluster 2 emerged as the optimal cluster, as indicated by three data points with a >50% probability of uncertainty out of the total 1133 members in Cluster 2. Consequently, our focus shifted toward investigating Cluster 2 in greater detail. 

### 3.8. The Optimal Cluster

Among the 55 identified EMT-related genes, 24 fell within Cluster 2, the optimal cluster. The final column of Table 1 delineates the assignment of EMT-related genes to each respective cluster. 

### 3.9. Biological Validation of Mclust Clustering Approach

We curated dataset D1, comprising 170 genes selected based on a significance threshold (*p*-value < 0.05) within our optimal cluster. These *p*-values reflect the differential expression levels of these genes between HTB and LTB conditions. We utilized Enrichr with D1, specifying the platform option as MSigDB Hallmark 2020, to validate the effectiveness of our clustering approach in representing biological significance. As detailed in Table 2, our dataset D1 exhibits a strong association with the EMT pathway, indicating that our clustering approach accurately preserves the biological significance of the EMT-related gene set. 

**Figure 3:**
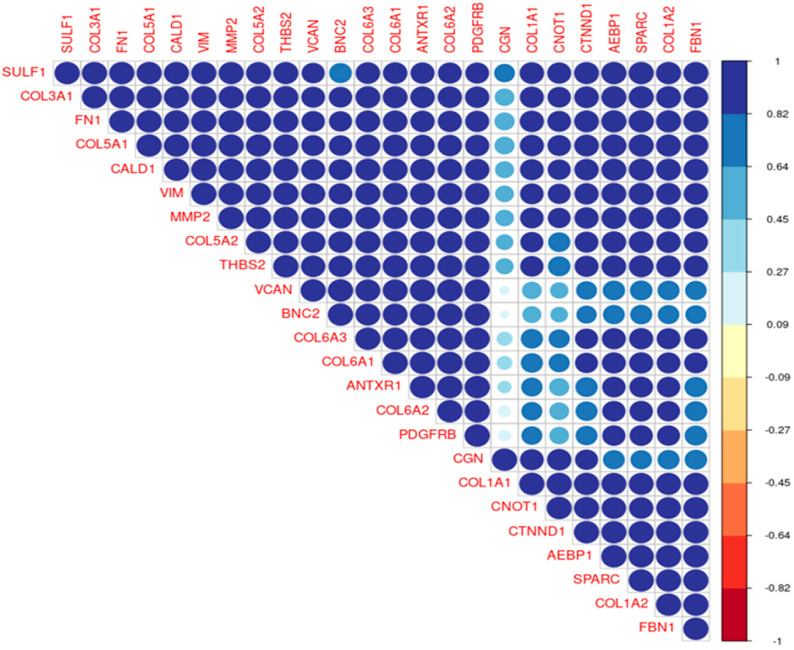
Correlation plot of 24 EMT-related genes in optimal cluster. Genes with strong positive correlations are depicted in dark blue (value close to 1) using dataset D2. And the larger circle indicates higher correlation. Larger circles indicate higher correlation levels.

In addition, we utilized a dataset denoted as D2, encompassing 24 genes associated with EMT within the optimal cluster to analyze their correlation. In Figure 3, dark blue denotes a positive correlation, while red indicates a negative correlation. It is observed that the majority of the 24 genes exhibit strong positive correlations with each other. Specific correlation coefficients are provided in Figure S3.

### 3.10. The Nature of EMT-Related Genes

**Figure 4:**
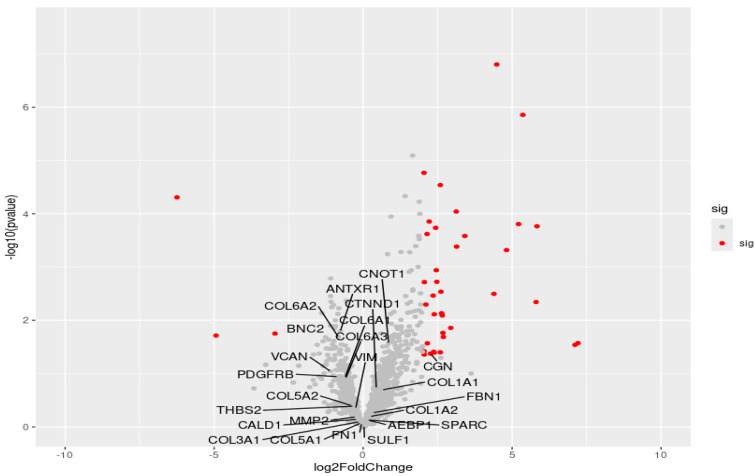
A volcano plot of the optimal cluster. The red dots indicate LFC > 2 and *p*-values < 0.05, denoted as “Sig” Only 24 EMT-related genes are labeled. The labeled genes exhibit minimal overlap with the “Sig” genes.

We aimed to examine the expression of D2 within our optimal cluster. Figure S4 illustrates the minimal disparity in the expression of these genes between HTB and LTB. These EMT-related genes might tend to be silenced, as evidenced by the volcano plot in Figure 4, where EMT-related genes are denoted by their respective gene names. Furthermore, genes labeled with “Sig” are highlighted in red, indicating an absolute log fold change (LFC) > 2 and *p*-values < 0.05. The labeled gene names correspond to EMT-related genes, with only a few overlapping, with the red dots signifying the filtering criteria of abs (LFC) > 2 and *p*-values < 0.05. Essentially, employing conventional filtering methods in this study might exclude nearly all EMT-related genes, causing erroneous conclusions.

### 3.11. Biological Interpretation

We employed two datasets for the subsequent pathway analysis: dataset D1, comprising 170 genes selected based on a significance threshold (*p*-value < 0.05) between HTB and LTB within our optimal cluster; dataset D2, including 24 genes associated with EMT from the optimal cluster. We utilized STRING [43], g:Profiler [44], ShineyGo [45], Pathway Commons [46], the GIPIA2 platform [47], and the Kaplan-Meier plotter [48] for this analysis.

#### 3.11.1. Pathway Analysis

**Figure 5:**
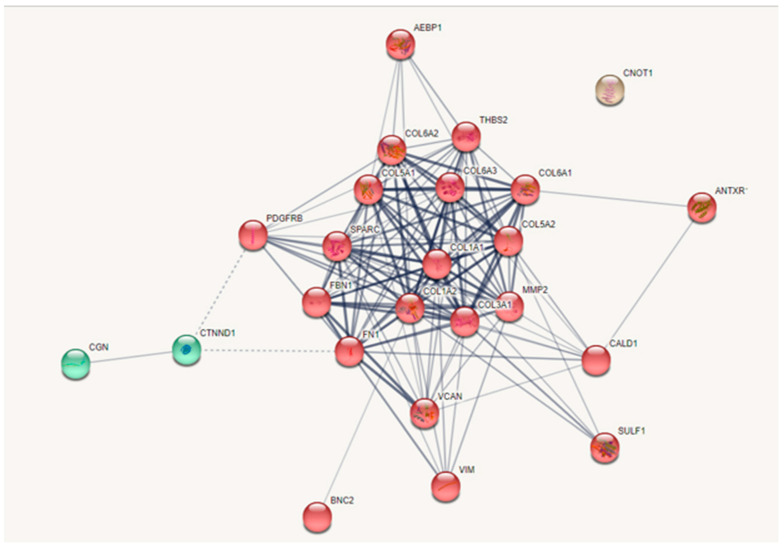
STRING pathway analysis of dataset D2. The pathway with the highest confidence score (0.9) in D2, comprising 24 EMT—associated genes from the optimal cluster.

Figure 5 illustrates the strong involvement of the collagen family in D2 when examined using STRING. Furthermore, Table S3 indicates a high association of D2 with the biological process (BP) of “extracellular matrix organization,” with a false discovery rate (FDR) of 8.87 × 10^−17^, as obtained from the STRING analysis. Additionally, the results obtained from the g:Profiler with D1 (Table S4) reinforce the significance of “ECM organization“, with a *p*-value of 8.051 × 10^−8^. Therefore, the EMT-related genes found in the optimal cluster are predominantly linked to the collagen family, suggesting their involvement in ECM-related biological processes.

#### 3.11.2. The Linkage between Collagen Type VI and MMP2

**Figure 6:**
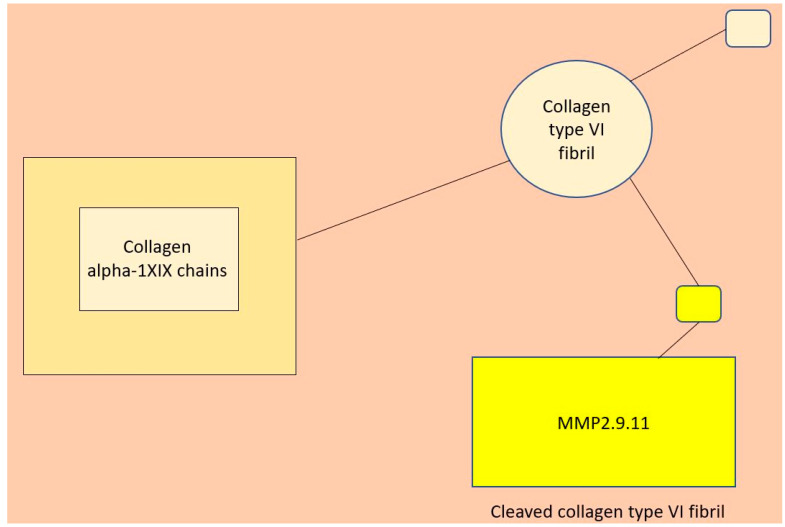
Biological linkage between *COL6A2* and *MMP2*, as depicted by Pathway Commons.

We utilized the ShineyGo and dataset D1 to pinpoint the specific collagen type. Table 3 displays the pathway enrichment analysis of D1, revealing the involvement of *COL6A2* in 4 out of 10 significant pathways identified. The pathways involving collagen type VI are highlighted in light green. Subsequently, we examined the association between *COL6A2* and specific matrix metalloproteinases (MMPs), particularly *MMP2*. Figure 5 suggests the presence of *MMP2* within the collagen family. Then, we utilized Pathway Commons [46] to investigate the interactions between the MMPs and type VI collagen. The results of this analysis are illustrated in Figure 6, with the relevant section highlighted by a red circle on the original (Figure S5).

#### 3.11.3. Top Five EMT-Related Genes and Validation with Public Data

In our optimal cluster, 5 (out of 24) genes displayed significance when comparing HTB and LTB groups. These genes included *COL6A2* (collagen type VI alpha 2 chain), *CNOT1* (CCR4-NOT transcription complex subunit 1), *BNC2* (Basonuclin Zinc Finger protein 2), *ANTXR1* (ANTXR cell adhesion molecule 1), and *CGN* (Cingulin), with *p*-values < 0.05. Specifically, their respective *p*-values were 0.021, 0.0271, 0.0219, 0.016, and 0.042. Table S5 provides detailed information pertaining to these genes from our dataset.

**Figure 7:**
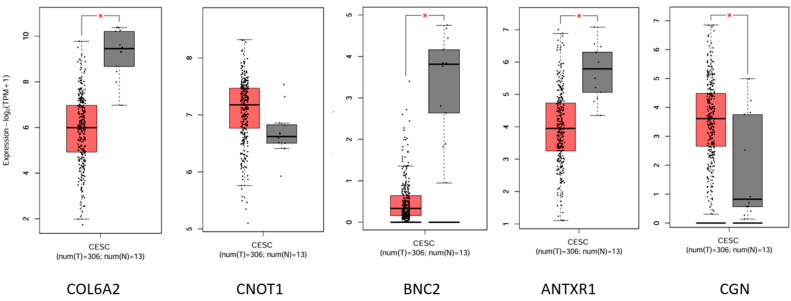
The different gene expressions of the top five EMT-related genes. The gene expression difference of the top five EMT-related genes was analyzed using the GIPIA2 platform. The disease code TCGA-CESC is depicted, with the gray box indicating a normal case and the brown-colored box representing a tumor case. A red asterisk (*) indicates a significance level of *p* < 0.05.

We utilized the GIPIA2 platform [47] to assess the expression levels associated with the disease code the Cancer Genome Atlas Cervical Squamous Cell Carcinoma and Endocervical Adenocarcinoma (TCGA-CESC) (Figure 7). We observed a decrease in the expression levels of *COL6A2*, *BNC2*, and *ANTXR* in tumors, while *CNOT1* and *CGN* exhibited contrasting behavior. This alignment is consistent with our observations (Table S5). 

**Figure 8:**
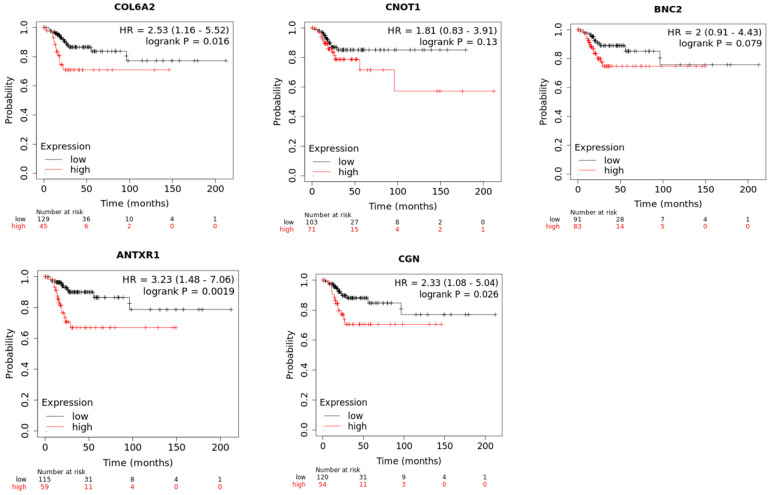
Kaplan-Meier analysis of RFS for top five EMT-related genes.

Additionally, we assessed the RFS of our top five genes using the Kaplan-Meier plotter [48], focusing on cervical squamous cell carcinoma. Kaplan-Meier plots for RFS of the top five genes (*COL6A2*, *CNOT1*, *BNC2*, *ANTXR1*, *CGN*) are presented in Figure 8, with *p*-values of 0.016, 0.13, 0.079, 0.0019, and 0.026, respectively. 

### 3.12. Validation of COL6A2 with Additional Clustering Algorithms 

To validate the biological findings of Mclust, particularly in identifying *COL6A2* or related biological processes (BPs), we employed seven additional clustering algorithms inspired by [51]. These algorithms included (1) hierarchical clustering with the Complete-linkage method (2) hierarchical clustering with the Ward.D method, (3) k-means, (4) DBSCAN, (5) KNN-based Louvain community detection, (6) hierarchical k-means, and (7) FANNY clustering. For consistency, we utilized a total of nine clusters and selected the cluster containing the highest number of EMT-related genes. The results, depicted in Figure S6, include pathway analyses of the EMT-related genes within each cluster for the different algorithms, with detailed BP results presented in the accompanying table. Table S6 presents the results of these additional seven clustering algorithms. In conclusion, all additional algorithms demonstrated the involvement of *COL6A2* and ECM-related pathways. 

## 4. Discussion

In this study, we utilized a large gene count matrix in RNA-seq analysis, which required meticulous preprocessing to downscale the matrix. While dimensionality reduction algorithms might represent a plausible approach, they often result in information loss, including the loss of gene names. Our study underscores the efficacy of leveraging clustering algorithms, particularly the Mclust, as a pragmatic alternative. This approach facilitated the creation of succinct yet informative clusters, thereby optimizing data representation.

The methodology we used provided significant discoveries. Utilizing the Mclust algorithm, we generated nine distinct clusters. Subsequently, we pinpointed an optimal cluster for further scrutiny based on the concept of “uncertainty,” as discussed earlier (Section 3.7). This approach allowed for the creation of a significant gene list. Upon calculating the EMT score, we identified 55 EMT-related genes overlapping with our entire data matrix, with 24 of these genes located within our optimal cluster. Subsequently, classical *p*-value filtering was applied to select the significant gene list of 170 candidates from the optimal cluster for pathway analysis, confirming EMT pathways. Our analysis revealed collagen family involvement, particularly collagen type VI, and its association with MMP2 within the ECM. Additionally, we identified the top five EMT-related genes and examined their expression trends in relation to RFS. 

Unlike previous studies of cervical cancer, we found a notable enrichment of the collagen family within the optimal cluster, as correlated with the TB classification. Our findings underscore the relevance of the collagen family in cervical cancer, particularly in the context of TB, EMT, and ECM degradation. This finding reveals the modus operandi of cervical cancer, since a critical step in the progression of cancer cells toward malignancy involves disrupting cell adhesion, facilitating their detachment from the primary tumor site. The transition, i.e., the EMT, is characterized by decreased cell adhesion and increased migratory capacity [52,53,54]. Cancer cells activate enzymes such as MMPs through TGFβ, facilitating ECM breakdown [55,56]. Given the collagen-rich composition of the ECM, increased collagen levels are often observed. The collagen family activates specific signaling pathways, such as PI3K/AKT, leading to a molecular E/N-cadherin switch. This process contributes to the formation of tumor budding (TB) and ultimately promotes tumor metastasis and progression [57]. While extensive research has explored the association of various collagen types, such as I, II, and IV, with cancer [58,59,60], the relationship between TB and the collagen family, particularly collage type VI, in cervical cancer has received little attention. To the best of our knowledge, only one study specifically explored cervical cancer and *COL6A1* [61]. Our current investigation sheds light on collagen type VI, especially *COL6A2*, as a potential prognostic predictor correlated with TB. This warrants further comprehensive examination. 

Our findings can be summarized as follows. First, the Mclust algorithm exhibited efficacy in our cervical cancer dataset, notwithstanding limitations in sample size, thus obviating the necessity for an exhaustive analysis of the entire gene count matrix. In our context, the clustering analysis adeptly captured biologically meaningful patterns. This approach might be particularly well suited for identifying genes that are inducible. EMT-related genes are a prime example. Second, we established a robust association between collagen type VI and TB in cervical cancer. This association holds promising potential for utilizing these factors as prognostic indicators for cervical cancer. 

In the realm of RNAseq analysis, initiating the process with a gene filtering procedure before embarking on pathway analysis is customary. This method involves scrutinizing criteria such as LFC and *p*-value disparities between control and treatment cohorts. Such an approach demonstrated efficacy in delineating pertinent gene subsets, particularly within specific cancer contexts or established paradigms within the field. However, as illustrated in Figure 5, EMT-related genes remain dormant until certain conditions in their microenvironment become conducive to their expression. Adhering to traditional filtering methods might inadvertently exclude EMT-related genes from the so-called significant gene set. In our case, although there were initially 24 EMT-related genes in the optimal cluster, only 5 EMT-related genes (*COL6A2*, *CNOT1*, *BNC2*, *ANTXR1*, *CGN*) remained after filtering with a significance threshold of a *p*-value < 0.05.

In our study, we utilized a clustering approach to enhance gene selection, moving beyond reliance solely on *p*-values or LFC. Although arguably, we realized that while selecting an appropriate clustering algorithm preserving biological significance poses a challenge, it is an essential step in gene selection. Specifically, this process allows for the analysis of a subset of genes rather than the entire gene count matrix, which can be substantial and often exceed 30,000 rows (genes), as in our case. Given the complexity of our dataset, we advocate the adoption of clustering algorithms that demonstrate superior performance in cervical cancer. 

Despite our significant findings linking collagen type VI to TB and identifying the top five genes, many opportunities for further refinement remain. Further elucidation of the exact relationship between these findings is warranted, particularly through more in-depth studies focusing on *COL6A2*, *CNOT1*, *BNC2*, *ANTXR1*, and *CGN*. Moreover, discerning whether a specific collagen type among the eight collagen genes (*COL1A1*, *COL1A2*, *COL3A1*, *COL5A1*, *COL5A2*, *COL6A1*, *COL6A2*, *COL6A3*) in the optimal cluster exerts a more pronounced influence in cervical cancer, as well as whether COL6A2 holds greater significance compared to others in this context, would be insightful. 

Due to the lack of normal data and the imbalance between HTB and LTB in our dataset, we validated our findings using TCGA data from a well-known platform. However, gene expression lacks consistency in RNAseq, and differences in study preparation, such as tissue type and the specific lesion samples taken, can lead to contrasting results. 

In addition, it is common for any type of tumor to disrupt the ECM and lead to a high proportion of collagen fragments. This aspect poses a significant challenge when choosing them as indicators of tumor invasiveness. Therefore, functional studies in the wet lab are necessary to confirm our findings. Furthermore, validation of the concordance in clinical settings is essential. However, our research made it possible to observe the trend of COL6A2 shown in Figure 7, showing a potential difference between HTB and LTB, which is intriguing and warrants further investigation in larger and more balanced TB cohorts. We are also conducting further investigations with spatial transcriptomics to follow up on the current study. 

## 5. Conclusions

We propose the utilization of clustering algorithms tailored to specific cancer types given the complexity of our dataset resulting from RNAseq analysis conducted on 20 cervical cancer tissue samples. Our study demonstrates the efficacy of the Mclust algorithm, particularly in cervical cancer analysis. Nearly half of the EMT-related genes were clustered together in our optimal cluster, highlighting the effectiveness of the algorithm among the nine clusters analyzed. Furthermore, our findings suggest a strong association between TB and prognosis, with collagen type VI emerging as a potential prognostic indicator in cervical cancer.

## Figures and Tables

**Table 1:** The clustering uncertainty from the Mclust. Column 2 displays the total number of members in each cluster. Column 3 indicates the count of members with a >50% probability of uncertainty. Column 4 presents the uncertainty percentage for each cluster, with the final column detailing the assignment of EMT-related genes to their respective clusters. The light green in Table 1 shows the optimal cluster 2.

Cluster	Members (*n*)	Uncertain Members (Probability > 50%) (*n*)	Uncertainty Percentage (%)	Number of EMT-Related Genes (*n)*
1	619	17	2.75	2
2	1133	3	0.26	24
3	2858	161	5.63	10
4	1952	119	6.10	6
5	3155	101	3.20	6
6	2798	198	7.08	3
7	602	12	1.99	3
8	904	18	1.99	1
9	1476	56	3.79	0
Total	15,497	685	32.79	55

**Table 2:** Enrichment analysis of dataset D1 in pathways identified by Enrichr. Pathways involving 170 genes (*p*-value < 0.05) within optimal cluster. Best enriched pathway is highlighted in light green.

Name	*p*-Value	Adjusted *p*-Value	Odds Ratio	Combined Score
Epithelial–mesenchymal transition	0.000000148	0.000006653	7.94	124.8
Glycolysis	0.00005505	0.001239	5.75	56.37
Interferon-gamma response	0.0003198	0.002878	5.05	40.65
E2F Targets	0.0003198	0.002878	5.05	40.65
p53 Pathway	0.0003198	0.002878	5.05	40.65
G2-M checkpoint	0.001642	0.01055	4.37	28.02
Myc targets V1	0.001642	0.01055	4.37	28.03
Adipogenesis	0.007353	0.03677	3.7	18.19
Apical junction	0.007353	0.03677	3.7	18.19
Protein secretion	0.009132	0.03871	5.17	24.28

**Table 3:** Enrichment of *COL6A2* in pathways identified by ShineyGo. Pathways involving collagen family VI in dataset D1 are highlighted in light green.

Pathway	Enrichment FDR	N Genes/Pathway Genes	Fold Enrichment	Genes
ECM–receptor interaction	0.00021204	7/88	11.23506	*COL6A2 HSPG2 ITGA2 ITGB4 LAMA4 LAMA5 LAMC2*
Apoptosis	0.0000747	9/136	9.346814	*TUBA1B CTSC CTSD CTSS IKBKB ITPR3 ATM LMNB2 TNFSF10*
Estrogen signaling pathway	0.000334375	8/138	8.187869	*CTSD HSPA8 HSP90AA1 ITPR3 KRT14 KRT16 KRT17 HSP90B1*
Biosynthesis of amino acids	0.027178002	4/74	7.634635	*ENO1 ALDOA PKM TALDO1*
Protein processing in the endoplasmic reticulum	0.006113592	7/169	5.850208	*HSPA8 HSP90AA1 SAR1B DNAJB11 UGGT1 HSP90B1 SEC24C*
Human papillomavirus infection	0.0000747	13/331	5.547219	*COL6A2 IKBKB ITGA2 ITGB4 LAMA4 LAMA5 LAMC2 MX1 ATM NFX1 NOTCH1 PKM FZD6*
Tight junction	0.02094193	6/169	5.014464	*TUBA1B AMOTL2 CGN EZR CACNA1D MYH14*
Focal adhesion	0.013140346	7/200	4.943426	*COL6A2 ITGA2 ITGB4 LAMA4 LAMA5 LAMC2 MYLK*
Pathways in cancer	0.000895628	14/530	3.730887	*CDKN2A CTNNA1 HSP90AA1 IKBKB IL6ST ITGA2 LAMA4 LAMA5 LAMC2 NOTCH1 PML TPM3 HSP90B1 FZD6*
PI3K-Akt signaling pathway	0.018364787	9/354	3.590866	*COL6A2 HSP90AA1 IKBKB ITGA2 ITGB4 LAMA4 LAMA5 LAMC2 HSP90B1*

## Data Availability

The data presented in this study are available on request from the corresponding author due to (specify the reason for the restriction).

## Supplementary Materials

The following supporting information can be downloaded at: https://www.mdpi.com/article/10.3390/life14081004/s1. Figure S1. The entire flow diagram of the current study. Figure S2. Parameterization of the covariance matrix of the Mclust. Figure S3. Correlation among 24 epithelial–mesenchymal transition (EMT)-related genes in optimal cluster. Figure S4. Gene expression difference of EMT-related 24 genes (dataset D2) in optimal cluster between HTB and LTH. Figure S5. Biological linkage between COL6A2 and MMP2, as depicted by Pathway Commons. Figure S6. STRING pathway analysis and biological process of 7 additional clustering algorithms. Table S1. Clinical variables of 20 cervical cancer samples. Table S2. Statistical test for five clustering methods. Table S3. The STRING results of the biological process in dataset D2 for the 24 EMT-related genes from the optimal cluster, with a 90% highest confidence score. Table S4. g:Profiler results of biological process in dataset D1. Table S5. Top five EMT-related genes. Table S6. The result of the additional seven clustering algorithms.
