# Supplementary material for: The Mclust Analysis of Tumor Budding Unveils the Role of the Collagen Family in Cervical Cancer Progression
Source: Life (Basel). 2024 Aug 13;14(8):1004. doi: 10.3390/life14081004 (PMC11355860; doi:10.3390/life14081004)
Supplement: Supplementary file 1 [file life-14-01004-s001.zip › life-3094442_Final_Supplememt_JUN222024_Olive.pptx]

## Slide 1
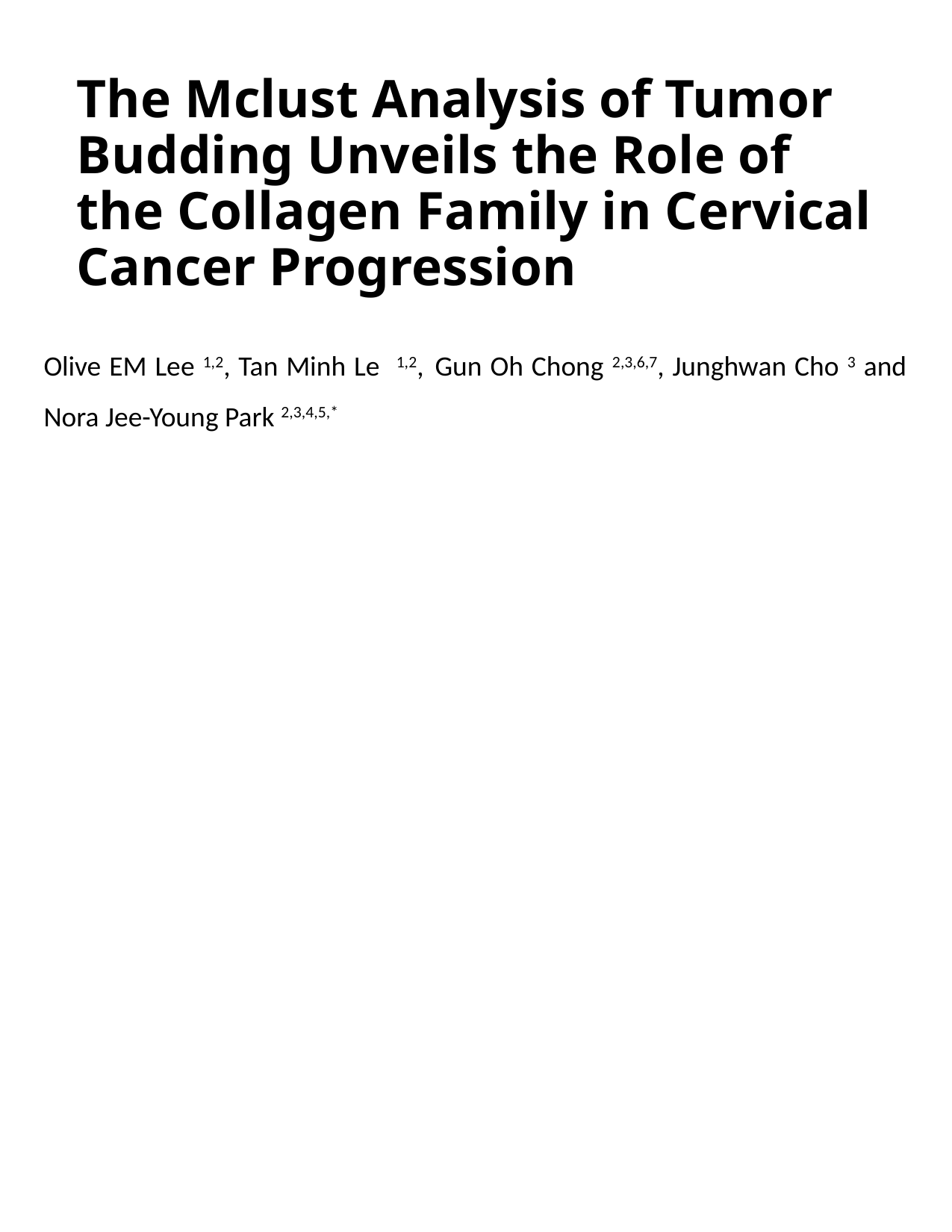

# The Mclust Analysis of Tumor Budding Unveils the Role of the Collagen Family in Cervical Cancer Progression
Olive EM Lee 1,2, Tan Minh Le 1,2, Gun Oh Chong 2,3,6,7, Junghwan Cho 3 and Nora Jee-Young Park 2,3,4,5,*

## Slide 2
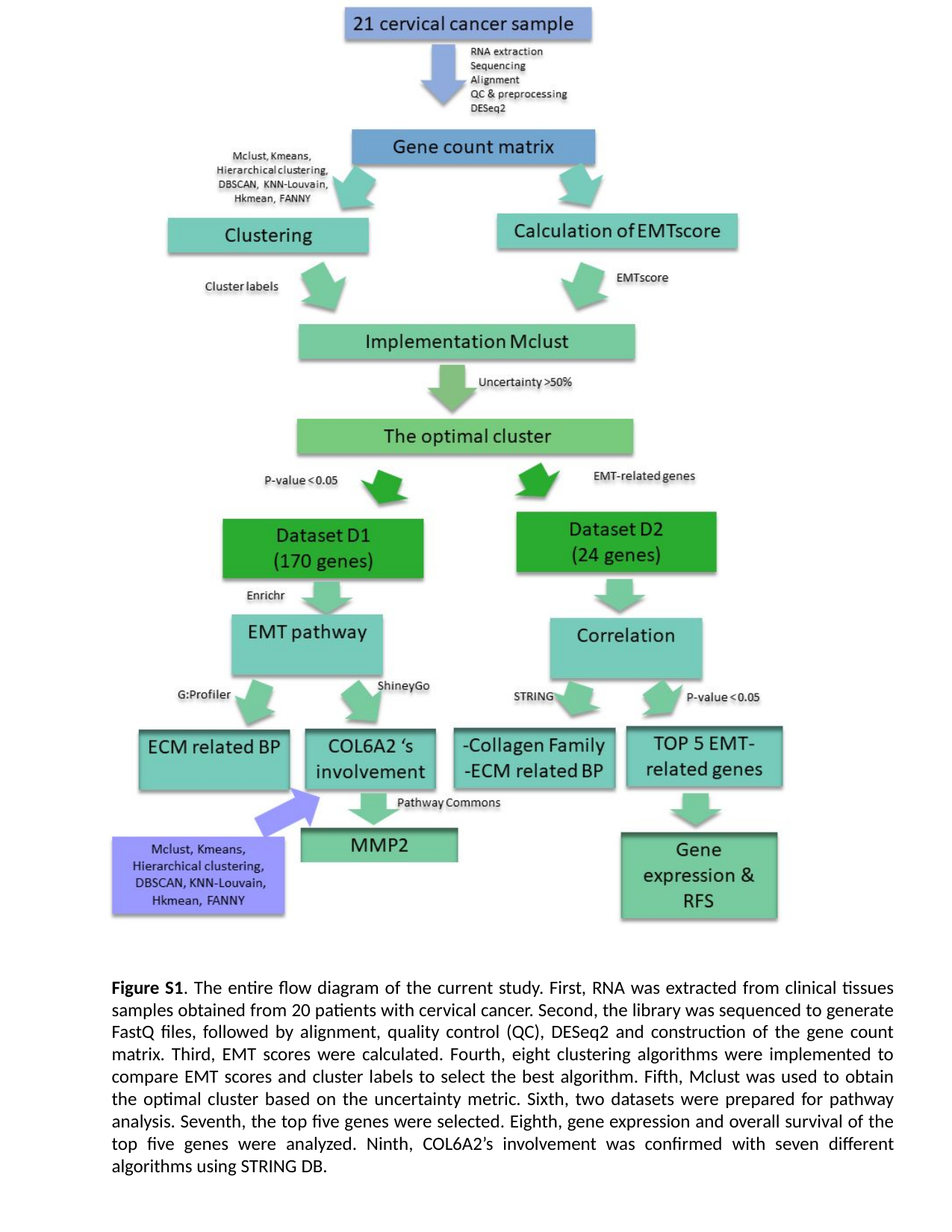

Figure S1. The entire flow diagram of the current study. First, RNA was extracted from clinical tissues samples obtained from 20 patients with cervical cancer. Second, the library was sequenced to generate FastQ files, followed by alignment, quality control (QC), DESeq2 and construction of the gene count matrix. Third, EMT scores were calculated. Fourth, eight clustering algorithms were implemented to compare EMT scores and cluster labels to select the best algorithm. Fifth, Mclust was used to obtain the optimal cluster based on the uncertainty metric. Sixth, two datasets were prepared for pathway analysis. Seventh, the top five genes were selected. Eighth, gene expression and overall survival of the top five genes were analyzed. Ninth, COL6A2’s involvement was confirmed with seven different algorithms using STRING DB.

## Slide 3
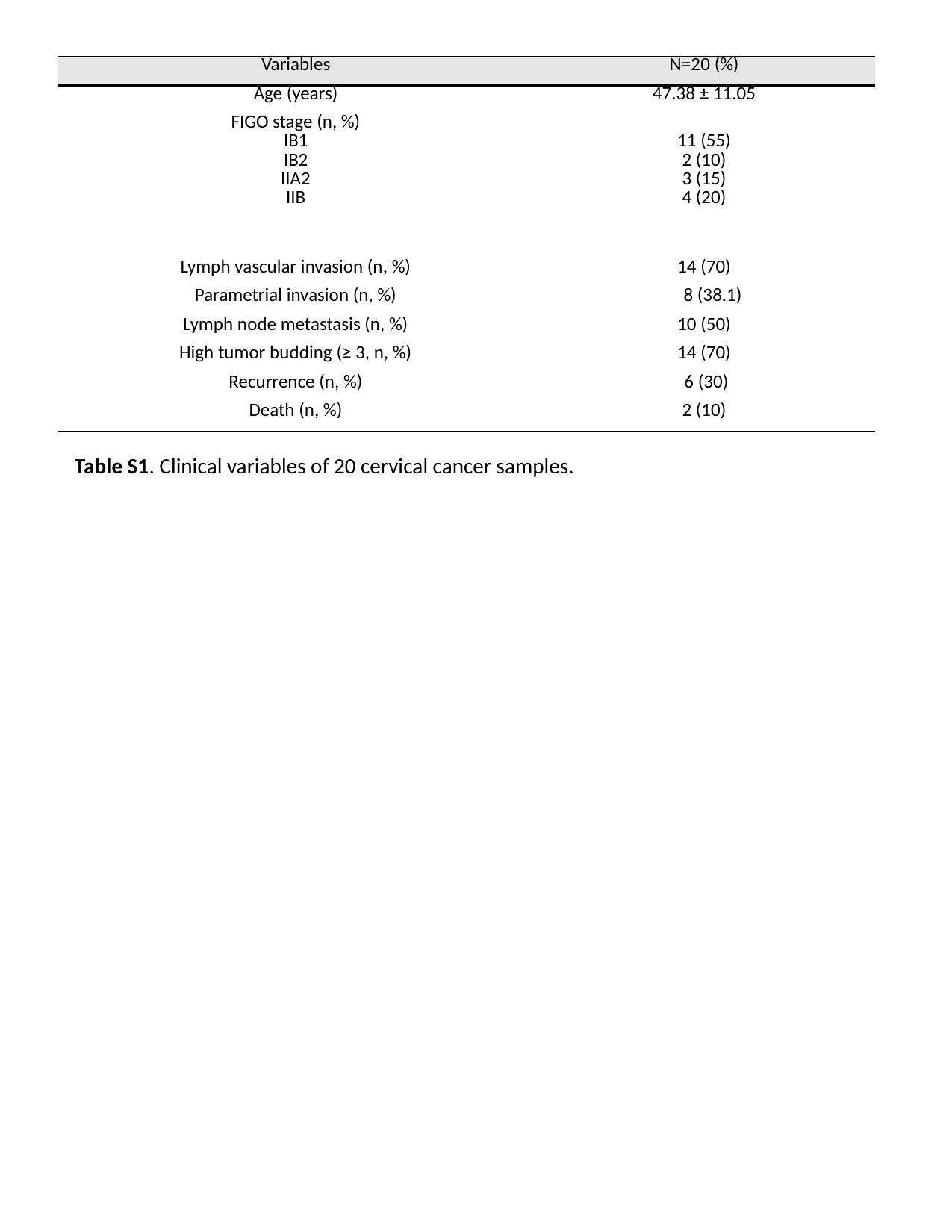

| Variables | N=20 (%) |
| --- | --- |
| Age (years) | 47.38 ± 11.05 |
| FIGO stage (n, %) IB1 IB2 IIA2 IIB | 11 (55) 2 (10) 3 (15) 4 (20) |
| Lymph vascular invasion (n, %) | 14 (70) |
| Parametrial invasion (n, %) | 8 (38.1) |
| Lymph node metastasis (n, %) | 10 (50) |
| High tumor budding (≥ 3, n, %) | 14 (70) |
| Recurrence (n, %) | 6 (30) |
| Death (n, %) | 2 (10) |
 Table S1. Clinical variables of 20 cervical cancer samples.

## Slide 4
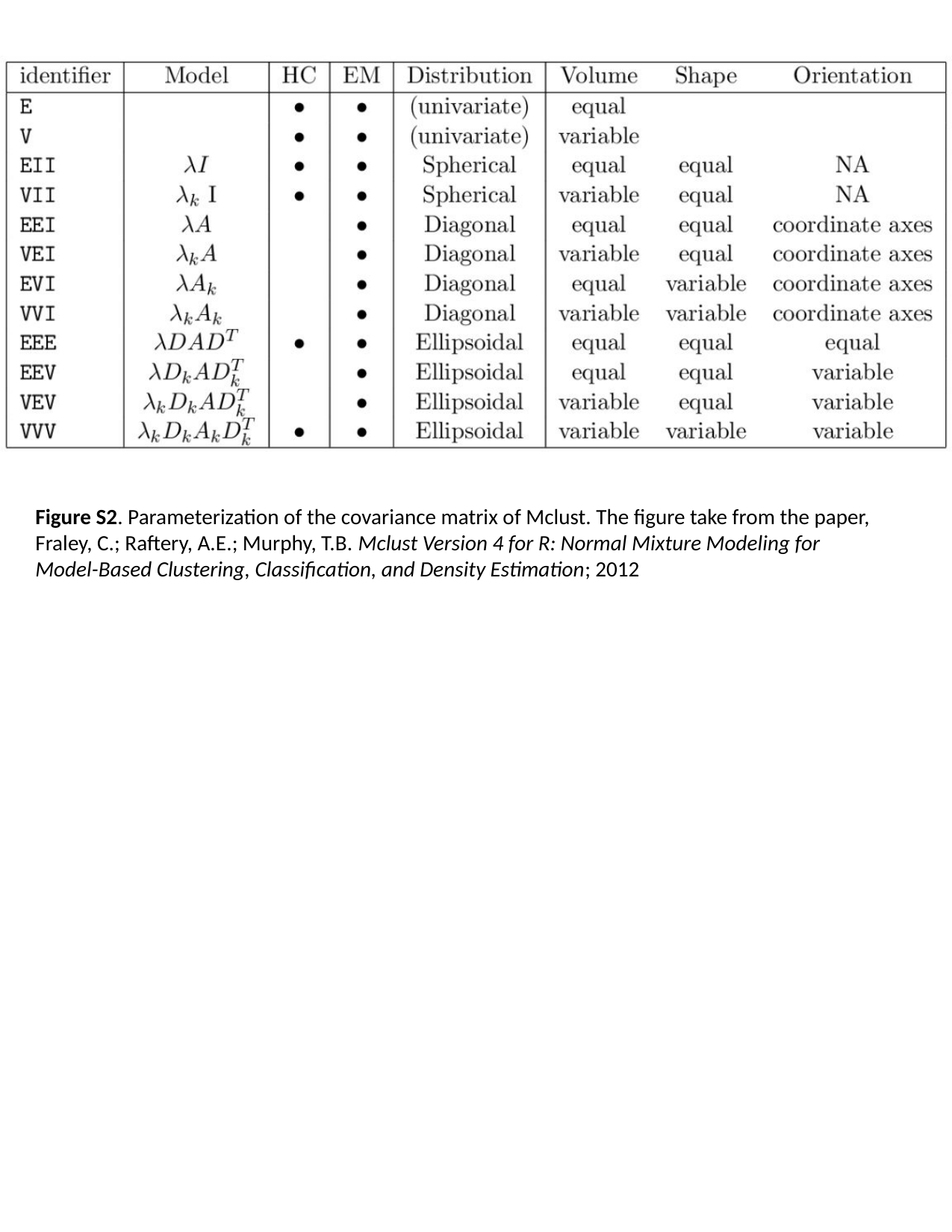

Figure S2. Parameterization of the covariance matrix of Mclust. The figure take from the paper,
Fraley, C.; Raftery, A.E.; Murphy, T.B. Mclust Version 4 for R: Normal Mixture Modeling for Model-Based Clustering, Classification, and Density Estimation; 2012

## Slide 5
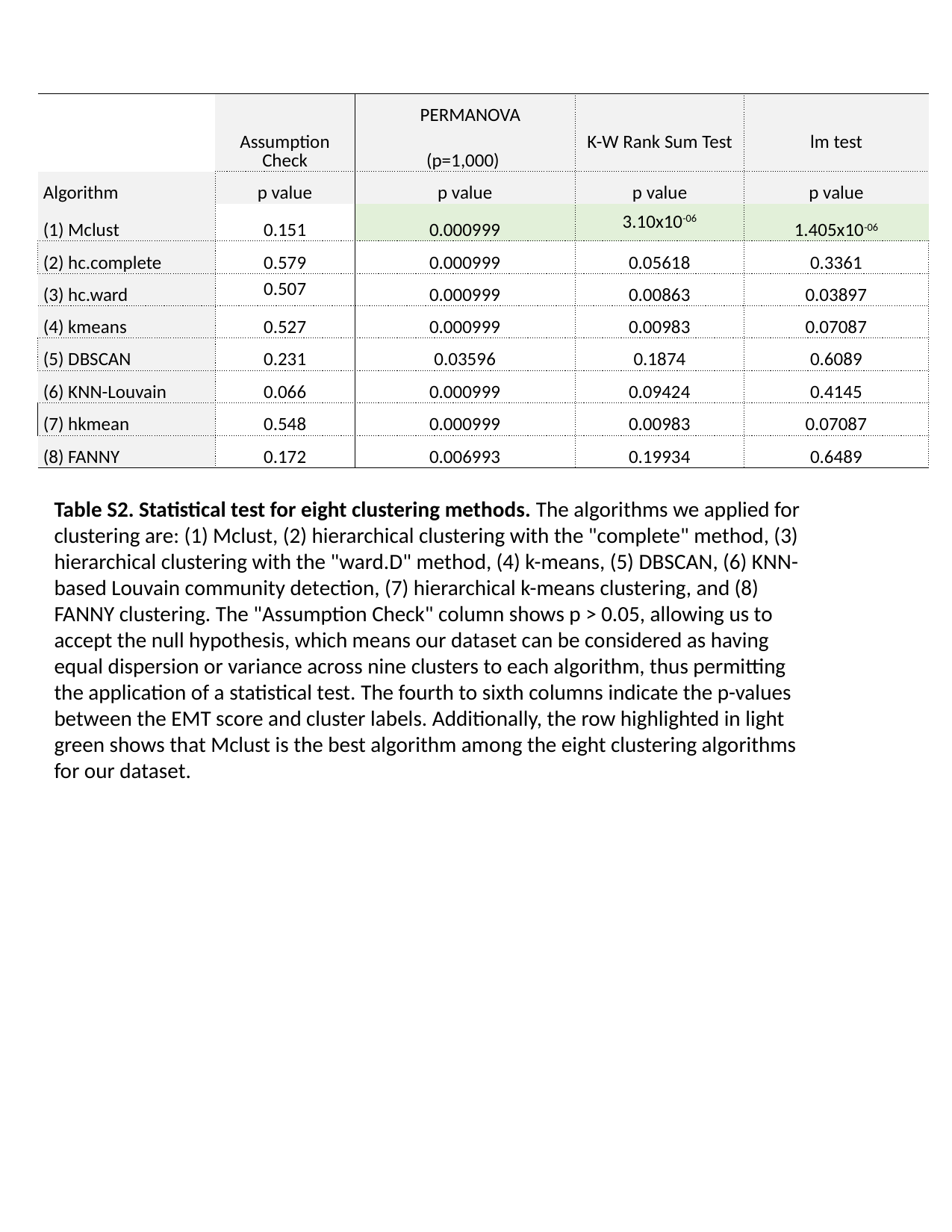

| | Assumption Check | PERMANOVA | K-W Rank Sum Test | lm test |
| --- | --- | --- | --- | --- |
| | | (p=1,000) | | |
| Algorithm | p value | p value | p value | p value |
| (1) Mclust | 0.151 | 0.000999 | 3.10x10^-06^ | 1.405x10^-06^ |
| (2) hc.complete | 0.579 | 0.000999 | 0.05618 | 0.3361 |
| (3) hc.ward | 0.507 | 0.000999 | 0.00863 | 0.03897 |
| (4) kmeans | 0.527 | 0.000999 | 0.00983 | 0.07087 |
| (5) DBSCAN | 0.231 | 0.03596 | 0.1874 | 0.6089 |
| (6) KNN-Louvain | 0.066 | 0.000999 | 0.09424 | 0.4145 |
| (7) hkmean | 0.548 | 0.000999 | 0.00983 | 0.07087 |
| (8) FANNY | 0.172 | 0.006993 | 0.19934 | 0.6489 |
Table S2. Statistical test for eight clustering methods. The algorithms we applied for clustering are: (1) Mclust, (2) hierarchical clustering with the "complete" method, (3) hierarchical clustering with the "ward.D" method, (4) k-means, (5) DBSCAN, (6) KNN-based Louvain community detection, (7) hierarchical k-means clustering, and (8) FANNY clustering. The "Assumption Check" column shows p > 0.05, allowing us to accept the null hypothesis, which means our dataset can be considered as having equal dispersion or variance across nine clusters to each algorithm, thus permitting the application of a statistical test. The fourth to sixth columns indicate the p-values between the EMT score and cluster labels. Additionally, the row highlighted in light green shows that Mclust is the best algorithm among the eight clustering algorithms for our dataset.

## Slide 6
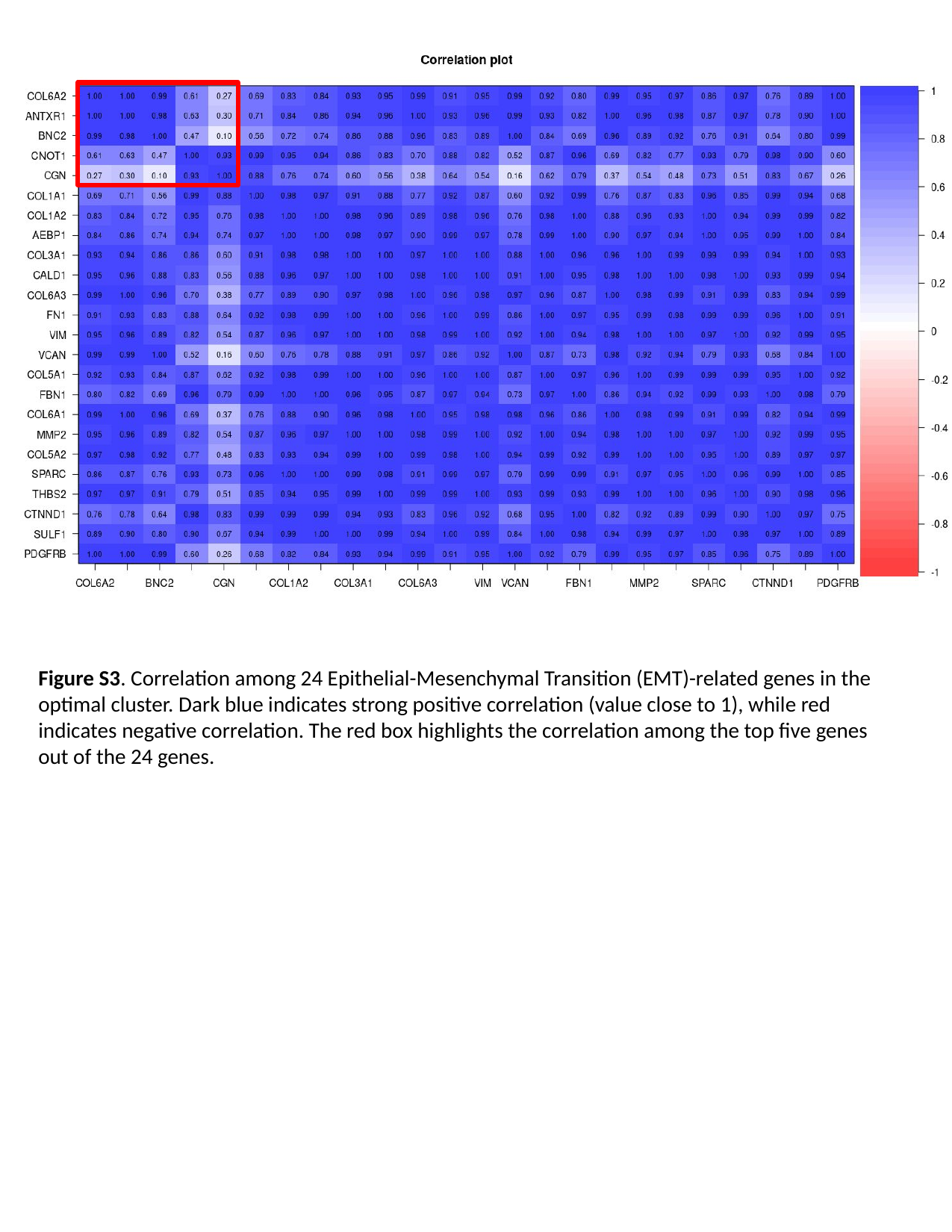

#
Figure S3. Correlation among 24 Epithelial-Mesenchymal Transition (EMT)-related genes in the optimal cluster. Dark blue indicates strong positive correlation (value close to 1), while red indicates negative correlation. The red box highlights the correlation among the top five genes out of the 24 genes.

## Slide 7
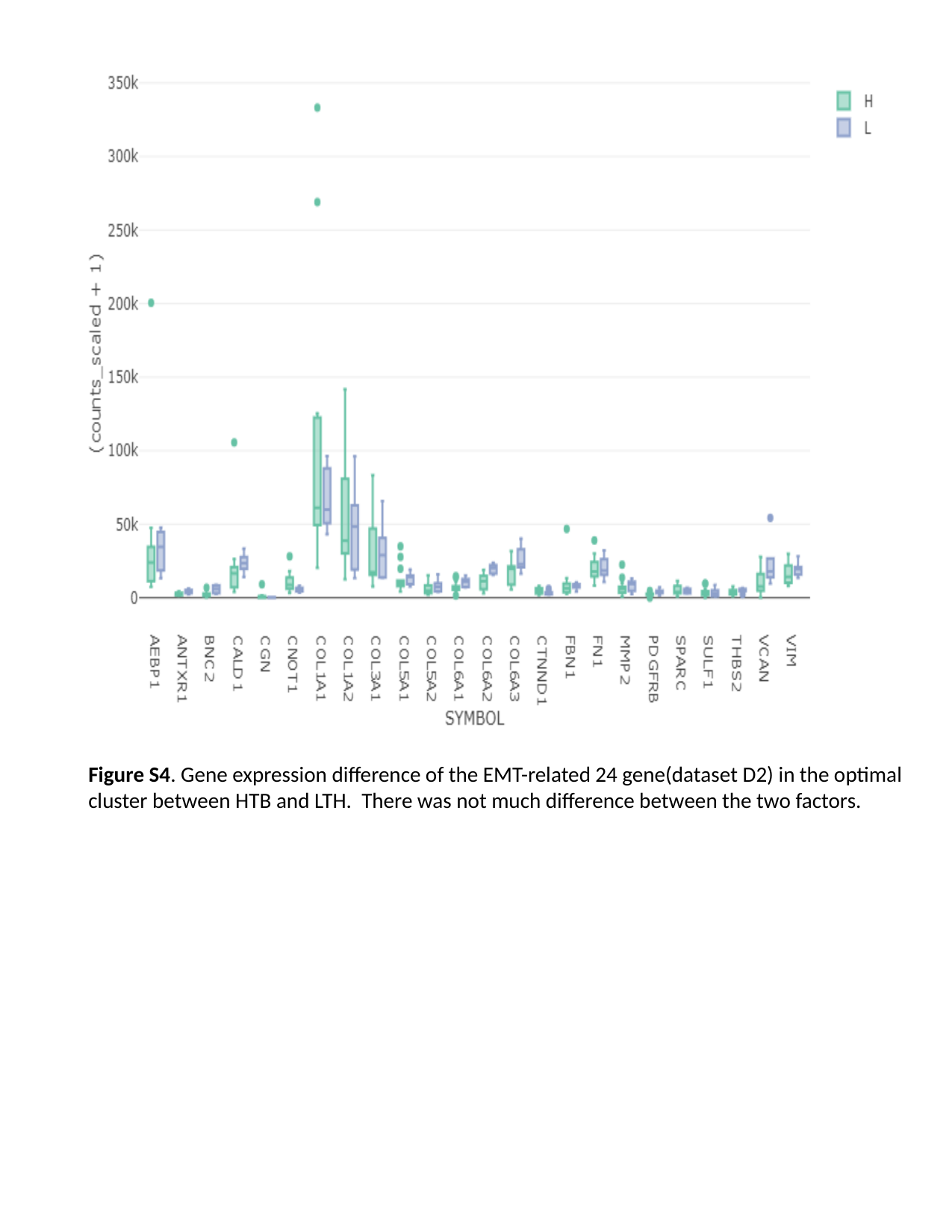

Figure S4. Gene expression difference of the EMT-related 24 gene(dataset D2) in the optimal cluster between HTB and LTH. There was not much difference between the two factors.

## Slide 8
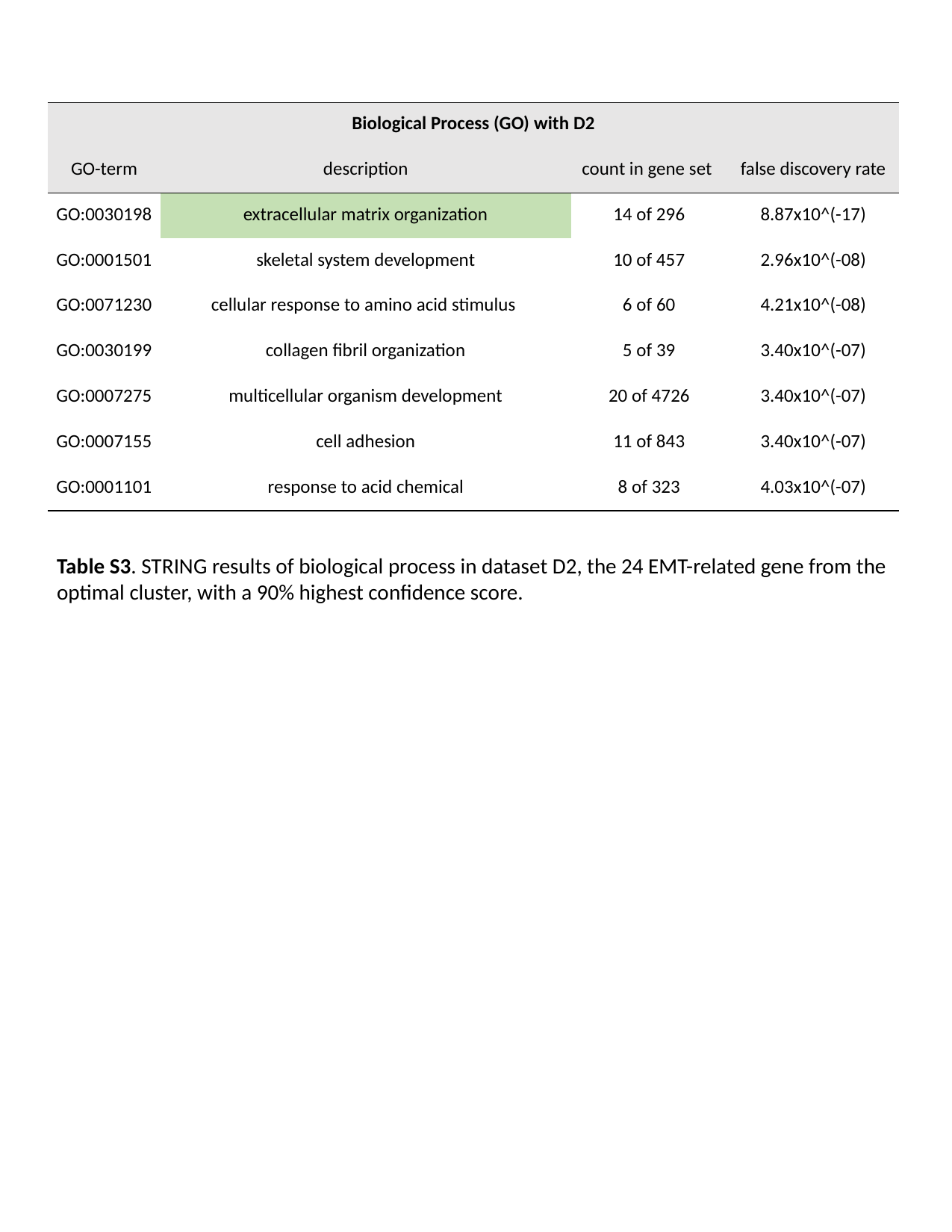

| Biological Process (GO) with D2 | | | |
| --- | --- | --- | --- |
| GO-term | description | count in gene set | false discovery rate |
| GO:0030198 | extracellular matrix organization | 14 of 296 | 8.87x10^(-17) |
| GO:0001501 | skeletal system development | 10 of 457 | 2.96x10^(-08) |
| GO:0071230 | cellular response to amino acid stimulus | 6 of 60 | 4.21x10^(-08) |
| GO:0030199 | collagen fibril organization | 5 of 39 | 3.40x10^(-07) |
| GO:0007275 | multicellular organism development | 20 of 4726 | 3.40x10^(-07) |
| GO:0007155 | cell adhesion | 11 of 843 | 3.40x10^(-07) |
| GO:0001101 | response to acid chemical | 8 of 323 | 4.03x10^(-07) |
Table S3. STRING results of biological process in dataset D2, the 24 EMT-related gene from the optimal cluster, with a 90% highest confidence score.

## Slide 9
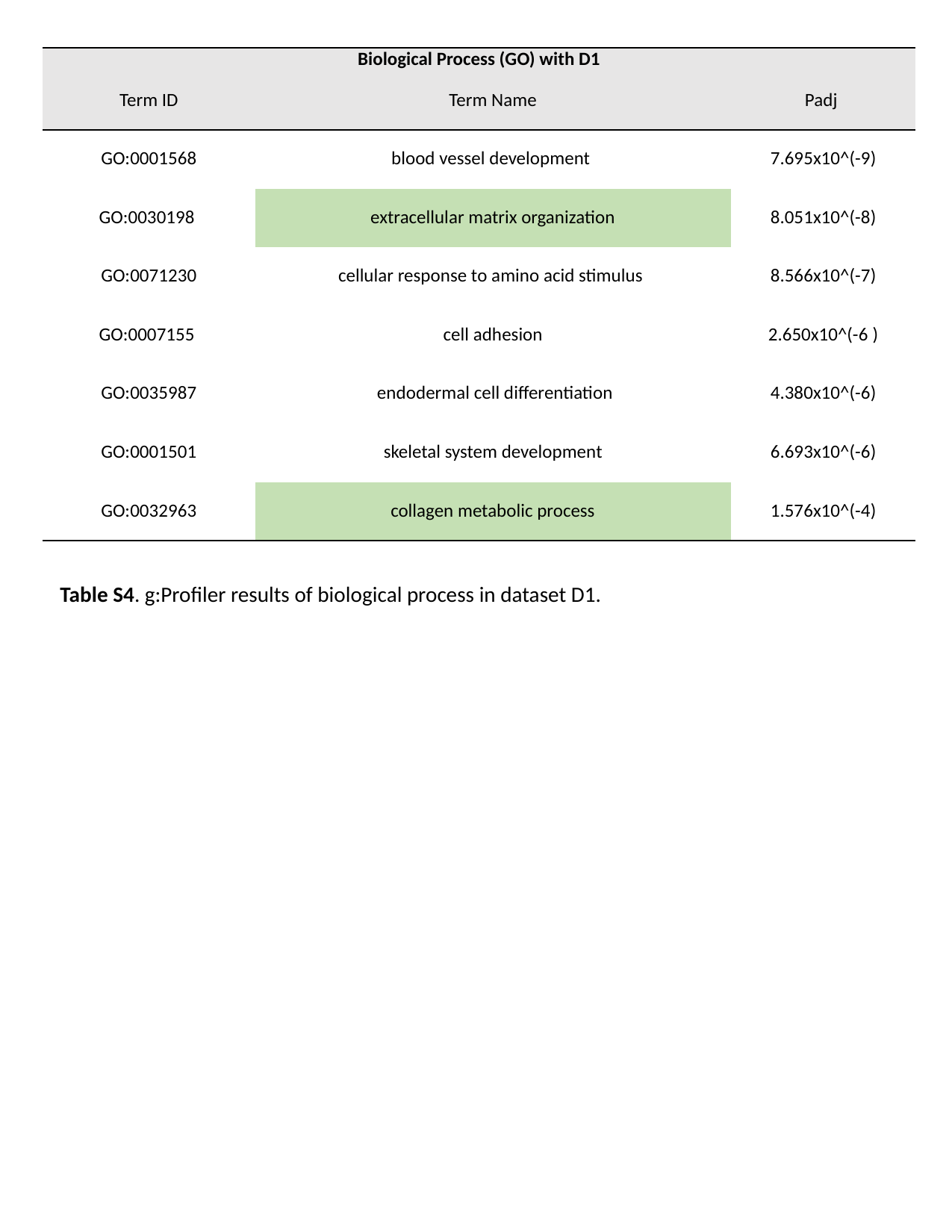

| Biological Process (GO) with D1 | | |
| --- | --- | --- |
| Term ID | Term Name | Padj |
| GO:0001568 | blood vessel development | 7.695x10^(-9) |
| GO:0030198 | extracellular matrix organization | 8.051x10^(-8) |
| GO:0071230 | cellular response to amino acid stimulus | 8.566x10^(-7) |
| GO:0007155 | cell adhesion | 2.650x10^(-6 ) |
| GO:0035987 | endodermal cell differentiation | 4.380x10^(-6) |
| GO:0001501 | skeletal system development | 6.693x10^(-6) |
| GO:0032963 | collagen metabolic process | 1.576x10^(-4) |
Table S4. g:Profiler results of biological process in dataset D1.

## Slide 10
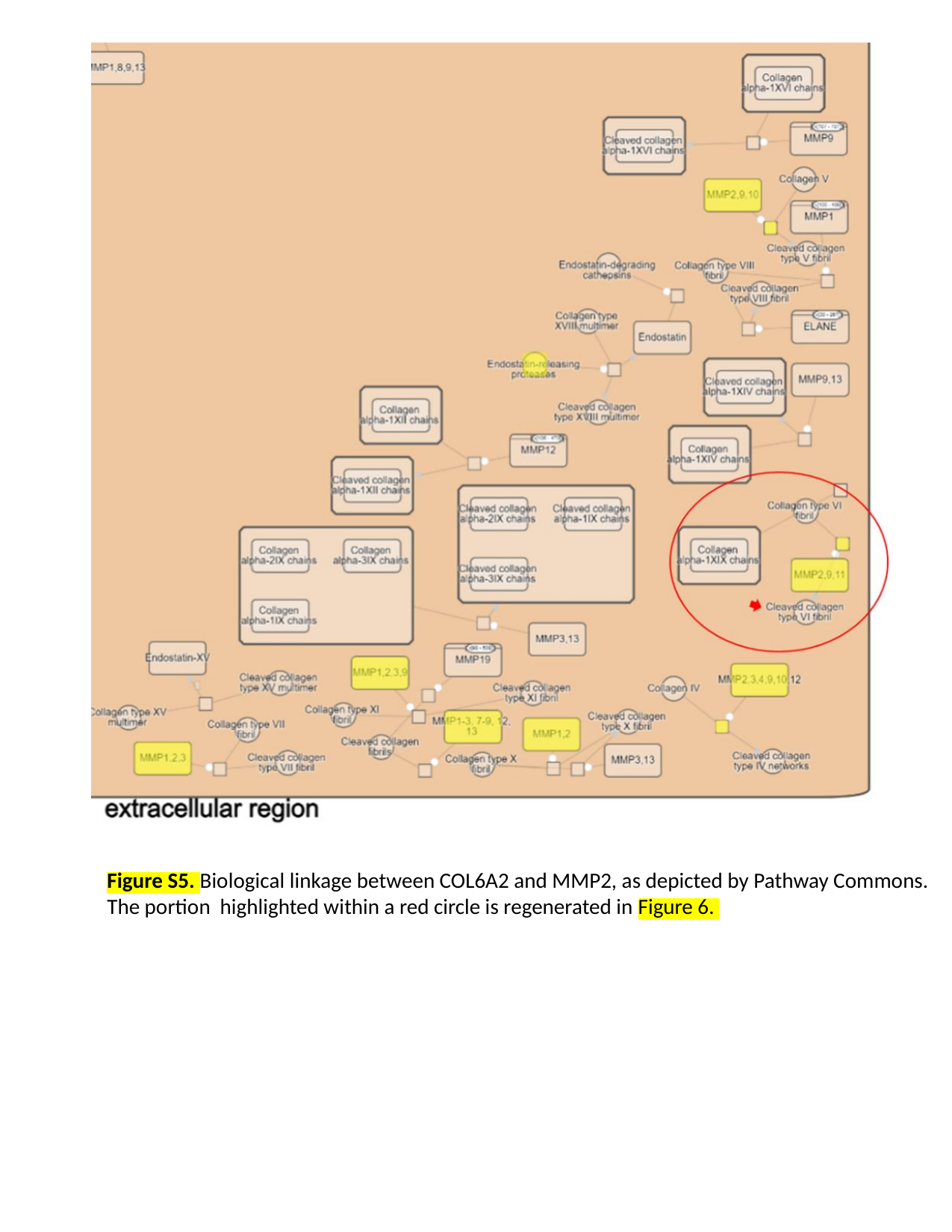

Figure S5. Biological linkage between COL6A2 and MMP2, as depicted by Pathway Commons.
The portion highlighted within a red circle is regenerated in Figure 6.

## Slide 11
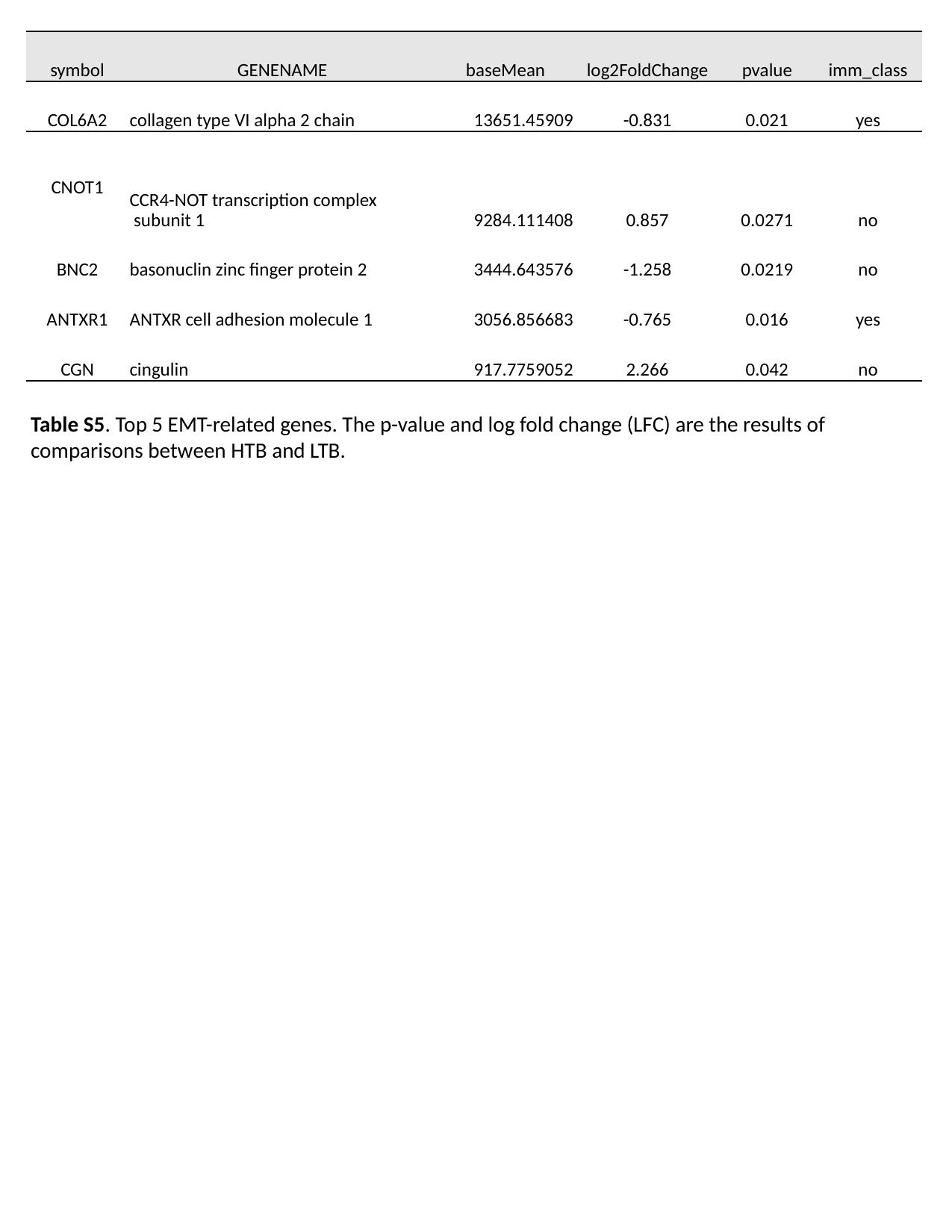

| symbol | GENENAME | baseMean | log2FoldChange | pvalue | imm\_class |
| --- | --- | --- | --- | --- | --- |
| COL6A2 | collagen type VI alpha 2 chain | 13651.45909 | -0.831 | 0.021 | yes |
| CNOT1 | CCR4-NOT transcription complex subunit 1 | 9284.111408 | 0.857 | 0.0271 | no |
| BNC2 | basonuclin zinc finger protein 2 | 3444.643576 | -1.258 | 0.0219 | no |
| ANTXR1 | ANTXR cell adhesion molecule 1 | 3056.856683 | -0.765 | 0.016 | yes |
| CGN | cingulin | 917.7759052 | 2.266 | 0.042 | no |
Table S5. Top 5 EMT-related genes. The p-value and log fold change (LFC) are the results of comparisons between HTB and LTB.

## Slide 12
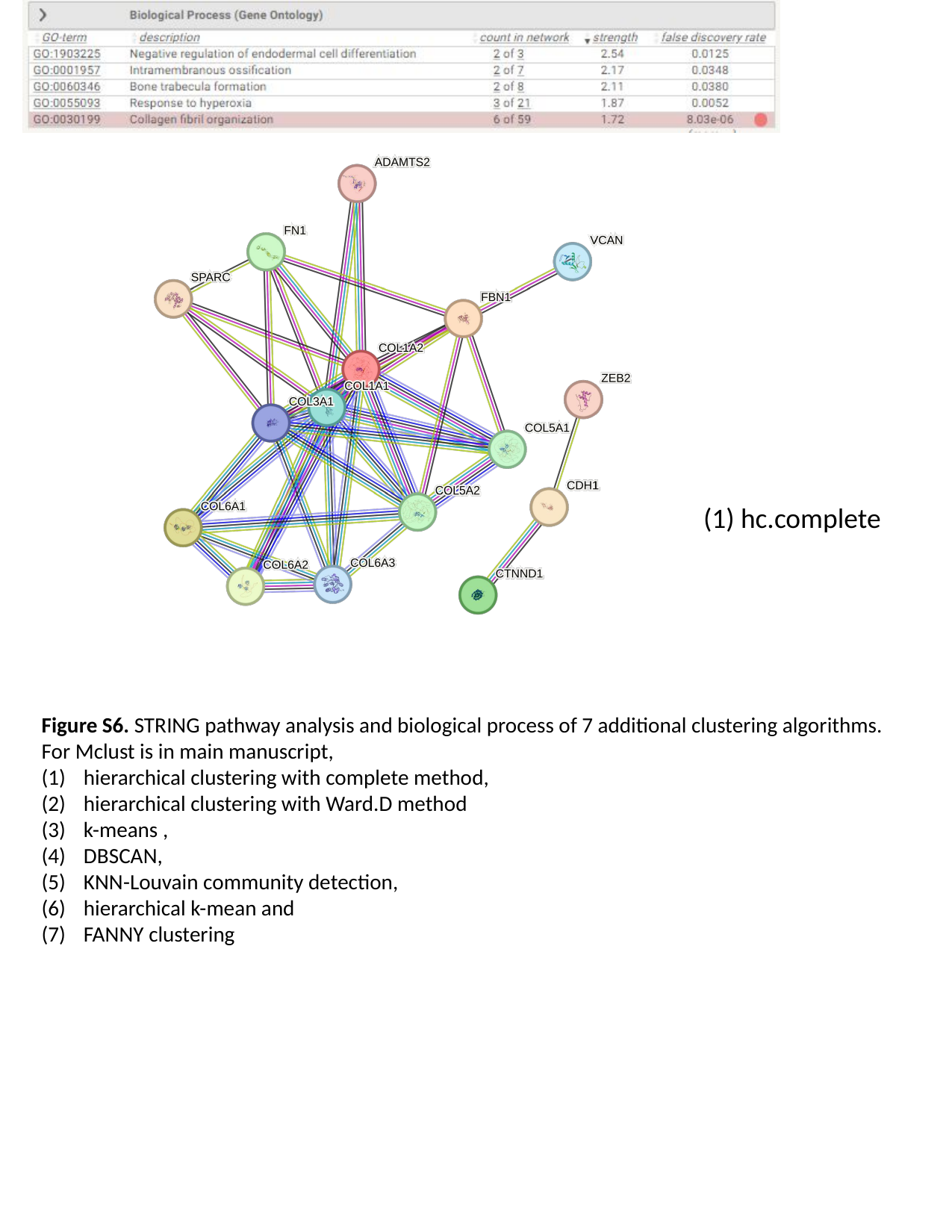

(1) hc.complete
Figure S6. STRING pathway analysis and biological process of 7 additional clustering algorithms.
For Mclust is in main manuscript,
hierarchical clustering with complete method,
hierarchical clustering with Ward.D method
k-means ,
DBSCAN,
KNN-Louvain community detection,
hierarchical k-mean and
FANNY clustering

## Slide 13
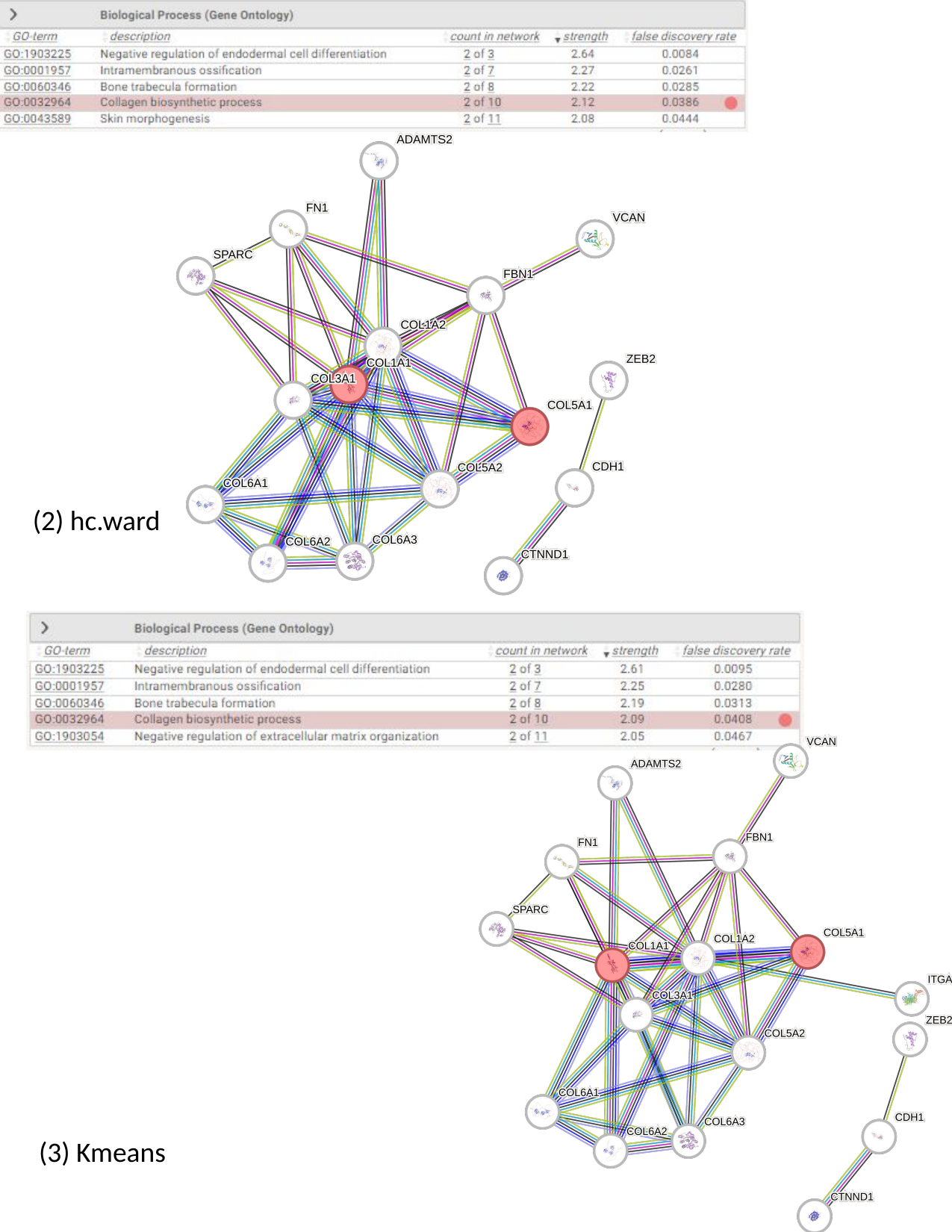

(2) hc.ward
(3) Kmeans

## Slide 14
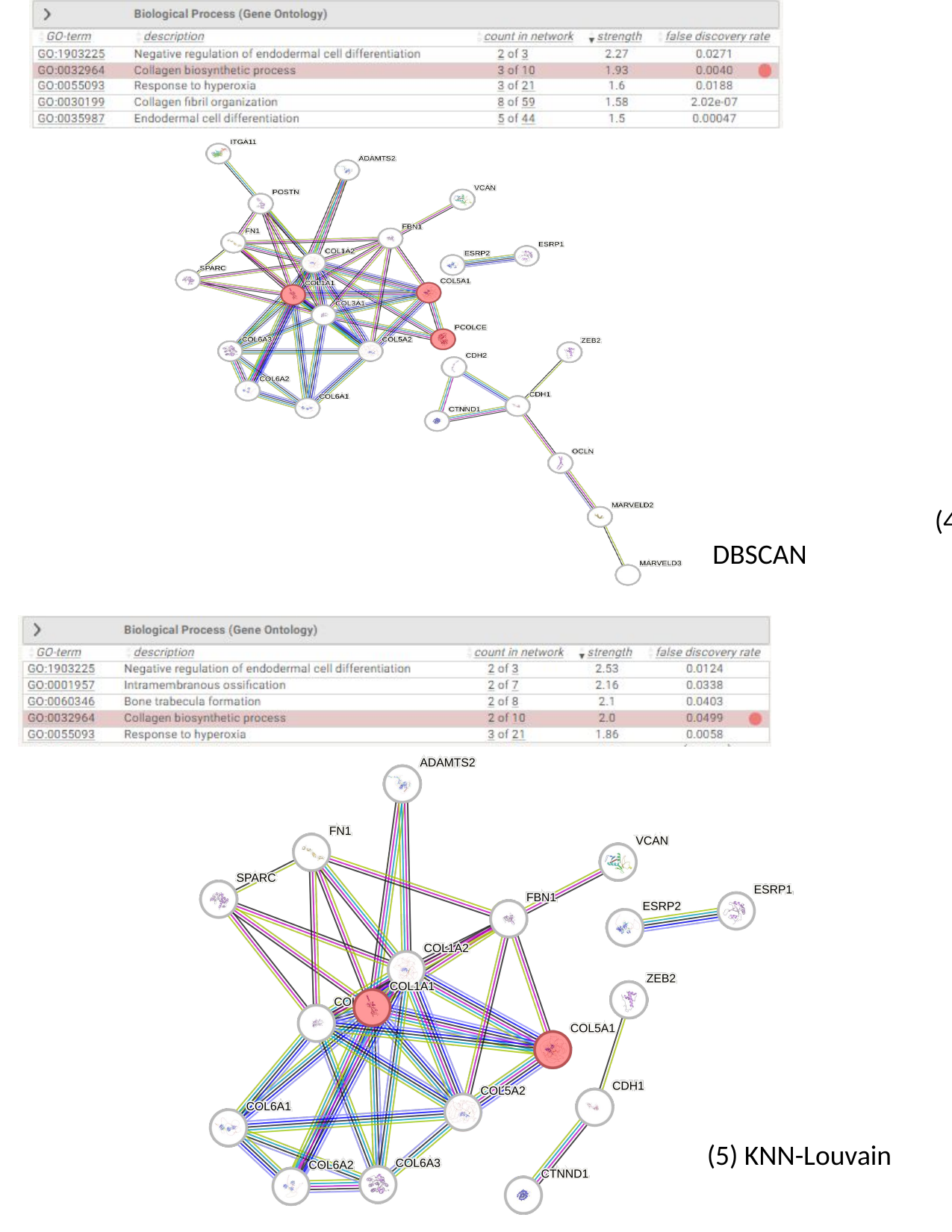

(4) DBSCAN
(5) KNN-Louvain

## Slide 15
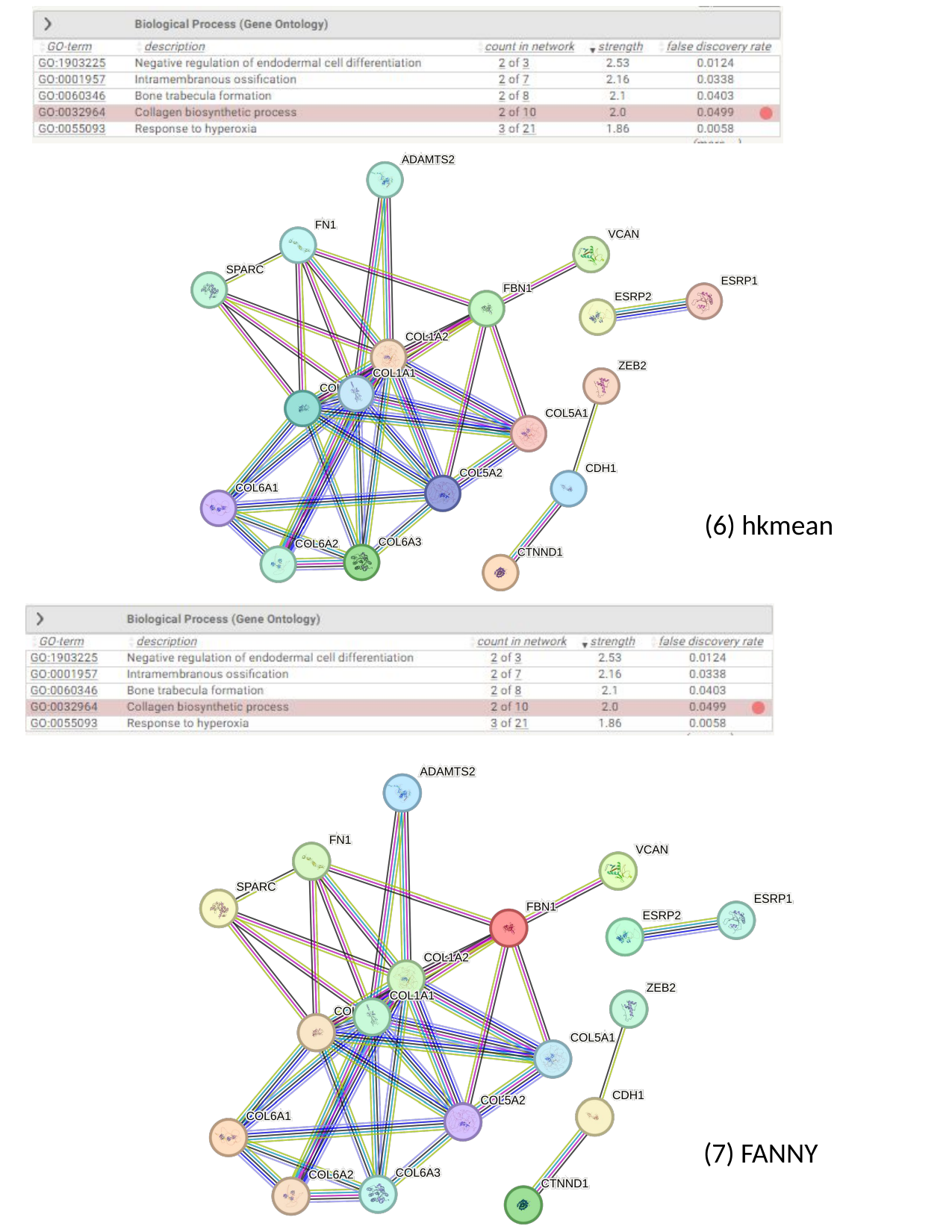

(6) hkmean
 (7) FANNY

## Slide 16
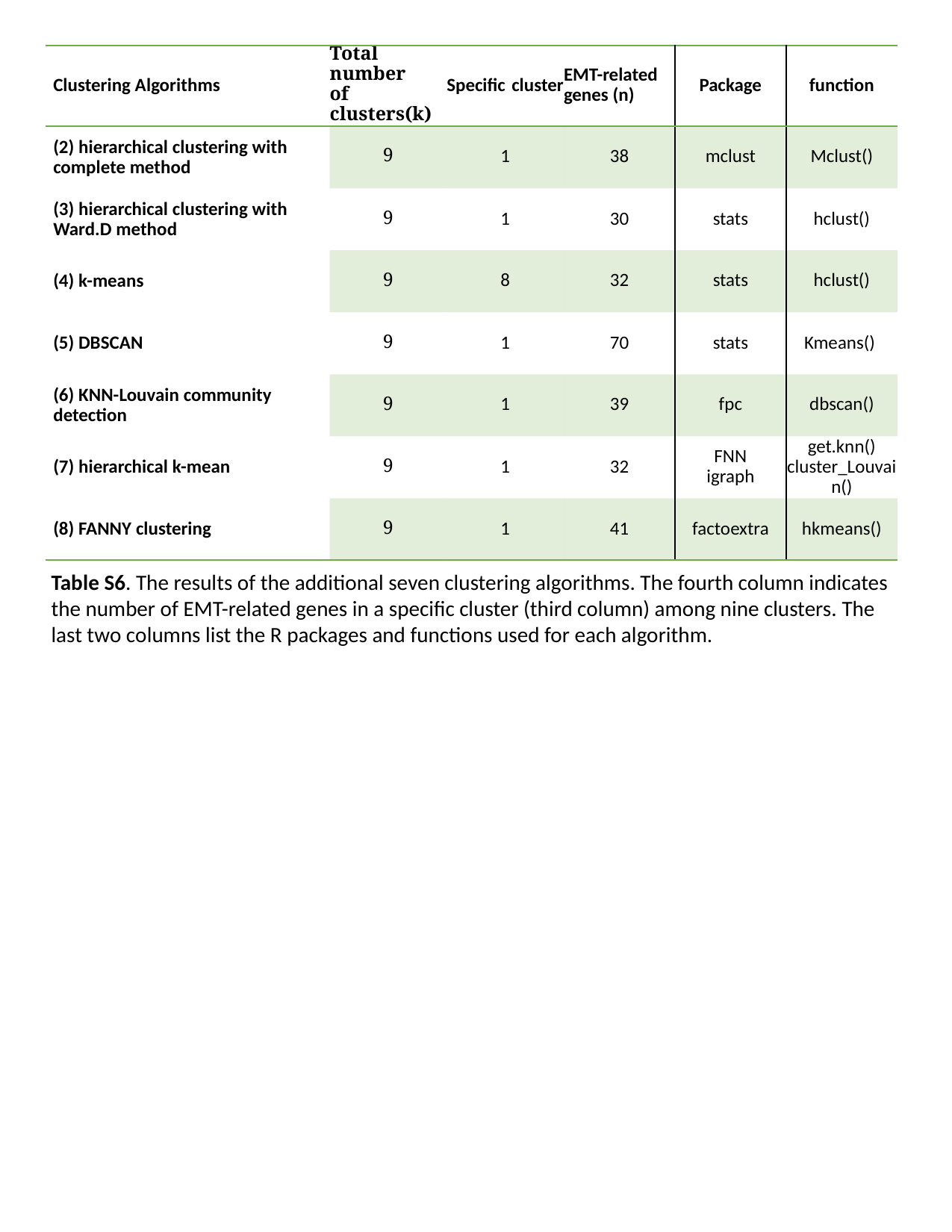

| Clustering Algorithms | Total number of clusters(k) | Specific cluster | EMT-related genes (n) | Package | function |
| --- | --- | --- | --- | --- | --- |
| (2) hierarchical clustering with complete method | 9 | 1 | 38 | mclust | Mclust() |
| (3) hierarchical clustering with Ward.D method | 9 | 1 | 30 | stats | hclust() |
| (4) k-means | 9 | 8 | 32 | stats | hclust() |
| (5) DBSCAN | 9 | 1 | 70 | stats | Kmeans() |
| (6) KNN-Louvain community detection | 9 | 1 | 39 | fpc | dbscan() |
| (7) hierarchical k-mean | 9 | 1 | 32 | FNN igraph | get.knn() cluster\_Louvain() |
| (8) FANNY clustering | 9 | 1 | 41 | factoextra | hkmeans() |
Table S6. The results of the additional seven clustering algorithms. The fourth column indicates the number of EMT-related genes in a specific cluster (third column) among nine clusters. The last two columns list the R packages and functions used for each algorithm.
