# Supplementary material for: The Mclust Analysis of Tumor Budding Unveils the Role of the Collagen Family in Cervical Cancer Progression
Source: Life (Basel). 2024 Aug 13;14(8):1004. doi: 10.3390/life14081004 (PMC11355860; doi:10.3390/life14081004)
Supplement: Supplementary file 1 [file life-14-01004-s001.zip › life-3094442_Supplement_JUL232024.pdf]

The Mclust Analysis of Tumor Budding Unveils the Role of the Collagen Family in Cervical Cancer Progression

Olive EM Lee [1,2], Tan Minh Le [1,2], Gun Oh Chong [2,3,6,7], Junghwan Cho [3] and Nora Jee-Young Park [2,3,4,5,*]

[1] Department of Biomedical Science, Graduate School, Kyungpook National University, Daegu 41944, Republic of Korea;
[2] BK21 Four Program, School of Medicine, Kyungpook National University, Daegu 41944, Republic of Korea;
[3] Clinical Omics Institute, Kyungpook National University, Daegu 41405, Republic of Korea;
[4] Department of Pathology, School of Medicine, Kyungpook National University, Daegu 41944, Republic of Korea;
[5] Department of Pathology, Kyungpook National University Chilgok Hospital, Daegu 41404, Republic of Korea;
[6] Department of Obstetrics and Gynecology, School of Medicine, Kyungpook National University, Daegu 41944, Republic of Korea;
[7] Department of Obstetrics and Gynecology, Kyungpook National University Chilgok Hospital, Daegu 41404, Republic of Korea

* Correspondence: pathpjy@knu.ac.kr (N.J.-Y.P.); 82-53-200-3405

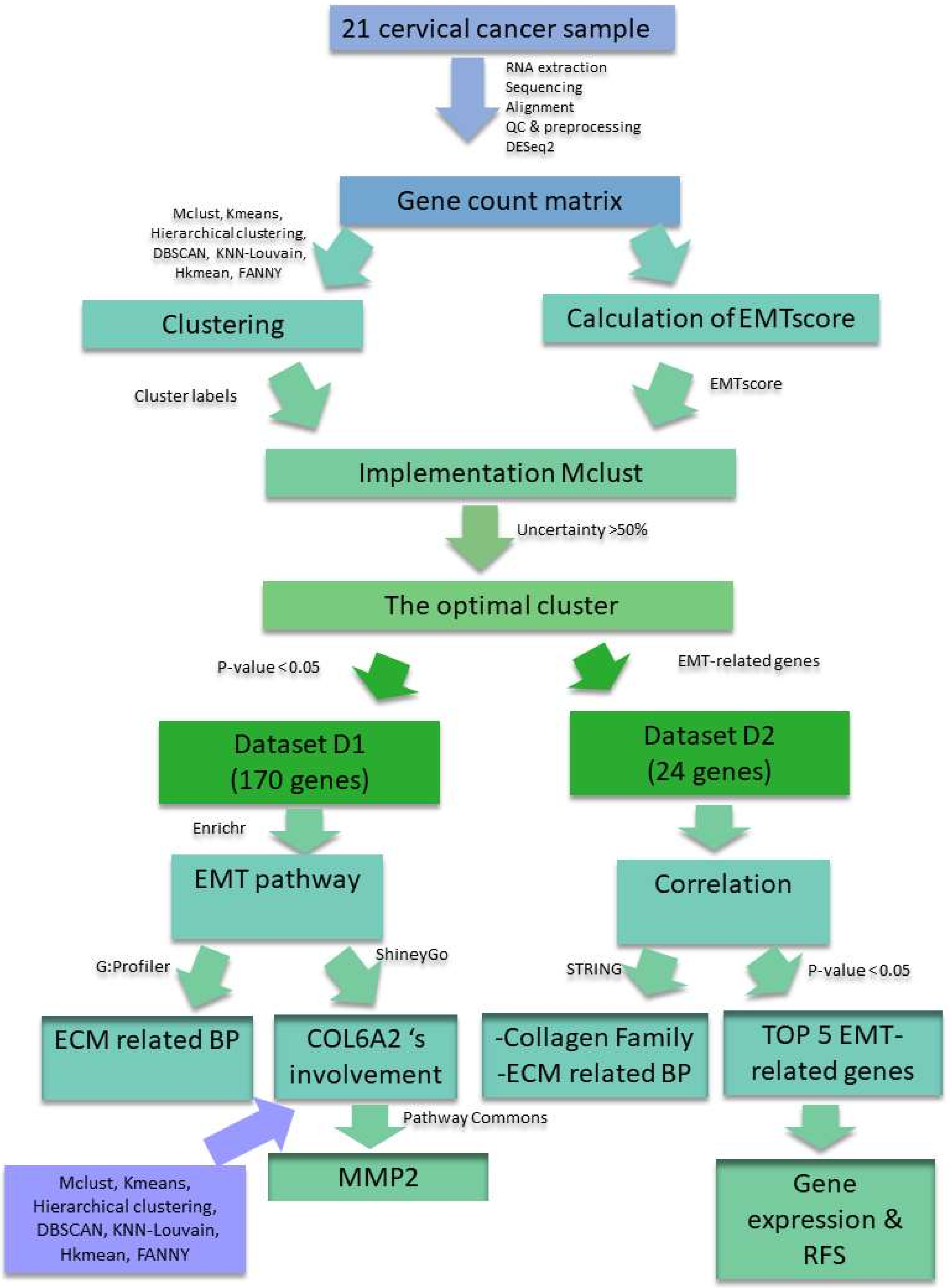

Figure S1. The entire flow diagram of the current study. First, RNA was extracted from clinical tissues samples obtained from 20 patients with cervical cancer. Second, the library was sequenced to generate FastQ files, followed by alignment, quality control (QC), DESeq2 and construction of the gene count matrix. Third, EMT scores were calculated. Fourth, eight clustering algorithms were implemented to compare EMT scores and cluster labels to select the best algorithm. Fifth, Mclust was used to obtain the optimal cluster based on the uncertainty metric. Sixth, two datasets were prepared for pathway analysis. Seventh, the top five genes were selected. Eighth, gene expression and overall survival of the top five genes were analyzed. Ninth, COL6A2's involvement was confirmed with seven different algorithms using STRING DB.

Variables	N=20 (%)
Age (years)	47.38 ± 11.05
FIGO stage (n, %)	
IB1	11 (55)
IB2	2 (10)
IIA2	3 (15)
IIB	4 (20)
Lymph vascular invasion (n, %)	14 (70)
Parametrial invasion (n, %)	8 (38.1)
Lymph node metastasis (n, %)	10 (50)
High tumor budding (≥ 3, n, %)	14 (70)
Recurrence (n, %)	6 (30)
Death (n, %)	2 (10)

Table S1. Clinical variables of 20 cervical cancer samples.

identifier	Model	HC	EM	Distribution	Volume	Shape	Orientation
E		•	•	(univariate)	equal		
V		•	•	(univariate)	variable		
EII	λI	•	•	Spherical	equal	equal	NA
VII	λ_k I	•	•	Spherical	variable	equal	NA
EEI	λA		•	Diagonal	equal	equal	coordinate axes
VEI	$\lambda_k A$		•	Diagonal	variable	equal	coordinate axes
EVI	λA_k		•	Diagonal	equal	variable	coordinate axes
VVI	$\lambda_k A_k$		•	Diagonal	variable	variable	coordinate axes
EEE	λDAD^T	•	•	Ellipsoidal	equal	equal	equal
EEV	$\lambda D_k A D_k^T$		•	Ellipsoidal	equal	equal	variable
VEV	$\lambda_k D_k A D_k^T$		•	Ellipsoidal	variable	equal	variable
VVV	$\lambda_k D_k A_k D_k^T$	•	•	Ellipsoidal	variable	variable	variable

Figure S2. Parameterization of the covariance matrix of Mclust. The figure take from the paper, Fraley, C.; Raftery, A.E.; Murphy, T.B. *Mclust Version 4 for R: Normal Mixture Modeling for Model-Based Clustering, Classification, and Density Estimation*; 2012

	Assumption Check	PERMANOVA (p=1,000)	K-W Rank Sum Test	lm test
Algorithm	p value	p value	p value	p value
(1) Mclust	0.151	0.000999	3.10×10^{-06}	1.405×10^{-06}
(2) hc.complete	0.579	0.000999	0.05618	0.3361
(3) hc.ward	0.507	0.000999	0.00863	0.03897
(4) kmeans	0.527	0.000999	0.00983	0.07087
(5) DBSCAN	0.231	0.03596	0.1874	0.6089
(6) KNN-Louvain	0.066	0.000999	0.09424	0.4145
(7) hkmean	0.548	0.000999	0.00983	0.07087
(8) FANNY	0.172	0.006993	0.19934	0.6489

Table S2. Statistical test for eight clustering methods. The algorithms we applied for clustering are: (1) Mclust, (2) hierarchical clustering with the "complete" method, (3) hierarchical clustering with the "ward.D" method, (4) k-means, (5) DBSCAN, (6) KNN-based Louvain community detection, (7) hierarchical k-means clustering, and (8) FANNY clustering. The "Assumption Check" column shows $p > 0.05$, allowing us to accept the null hypothesis, which means our dataset can be considered as having equal dispersion or variance across nine clusters to each algorithm, thus permitting the application of a statistical test. The fourth to sixth columns indicate the p-values between the EMT score and cluster labels. Additionally, the row highlighted in light green shows that Mclust is the best algorithm among the eight clustering algorithms for our dataset.

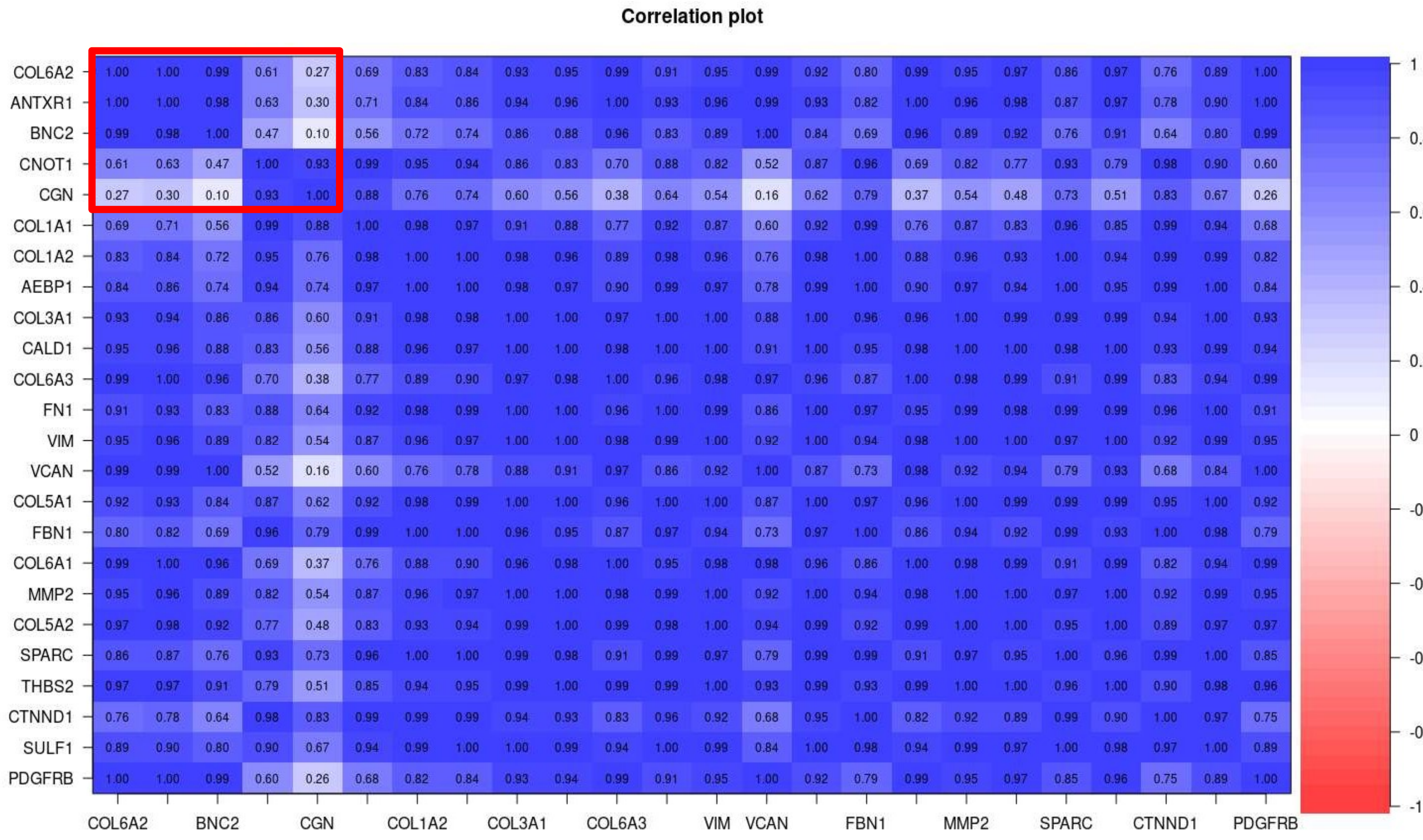

Figure S3. Correlation among 24 Epithelial-Mesenchymal Transition (EMT)-related genes in the optimal cluster. Dark blue indicates strong positive correlation (value close to 1), while red indicates negative correlation. The red box highlights the correlation among the top five genes out of the 24 genes.

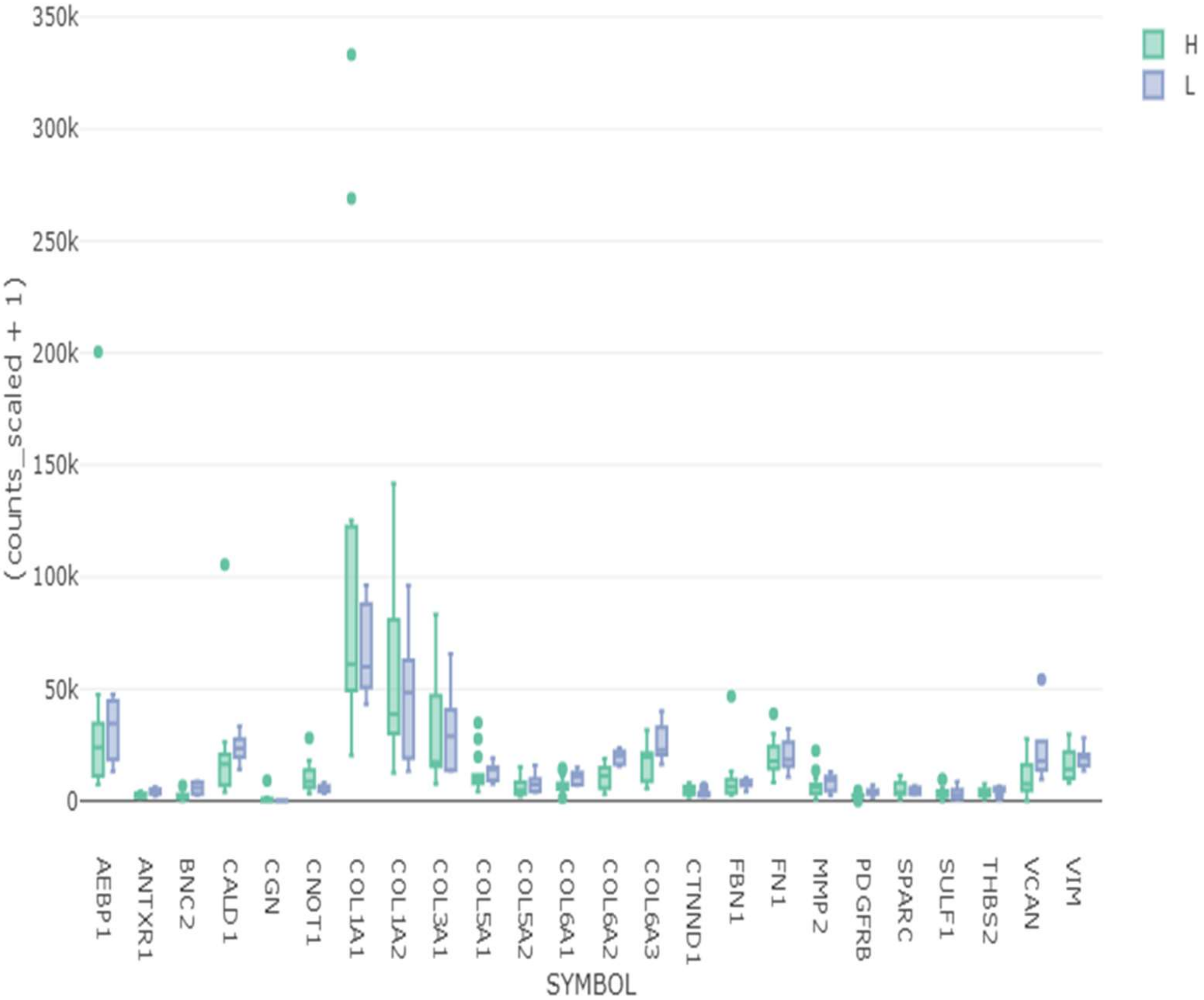

Figure S4. Gene expression difference of the EMT-related 24 gene(dataset D2) in the optimal cluster between HTB and LTH. There was not much difference between the two factors.

Biological Process (GO) with D2			
GO-term	description	count in gene set	false discovery rate
GO:0030198	extracellular matrix organization	14 of 296	8.87x10^(-17)
GO:0001501	skeletal system development	10 of 457	2.96x10^(-08)
GO:0071230	cellular response to amino acid stimulus	6 of 60	4.21x10^(-08)
GO:0030199	collagen fibril organization	5 of 39	3.40x10^(-07)
GO:0007275	multicellular organism development	20 of 4726	3.40x10^(-07)
GO:0007155	cell adhesion	11 of 843	3.40x10^(-07)
GO:0001101	response to acid chemical	8 of 323	4.03x10^(-07)

Table S3. STRING results of biological process in dataset D2, the 24 EMT-related gene from the optimal cluster, with a 90% highest confidence score.

Biological Process (GO) with D1		
Term ID	Term Name	Padj
GO:0001568	blood vessel development	7.695x10^(-9)
GO:0030198	extracellular matrix organization	8.051x10^(-8)
GO:0071230	cellular response to amino acid stimulus	8.566x10^(-7)
GO:0007155	cell adhesion	2.650x10^(-6)
GO:0035987	endodermal cell differentiation	4.380x10^(-6)
GO:0001501	skeletal system development	6.693x10^(-6)
GO:0032963	collagen metabolic process	1.576x10^(-4)

Table S4. g:Profiler results of biological process in dataset D1.

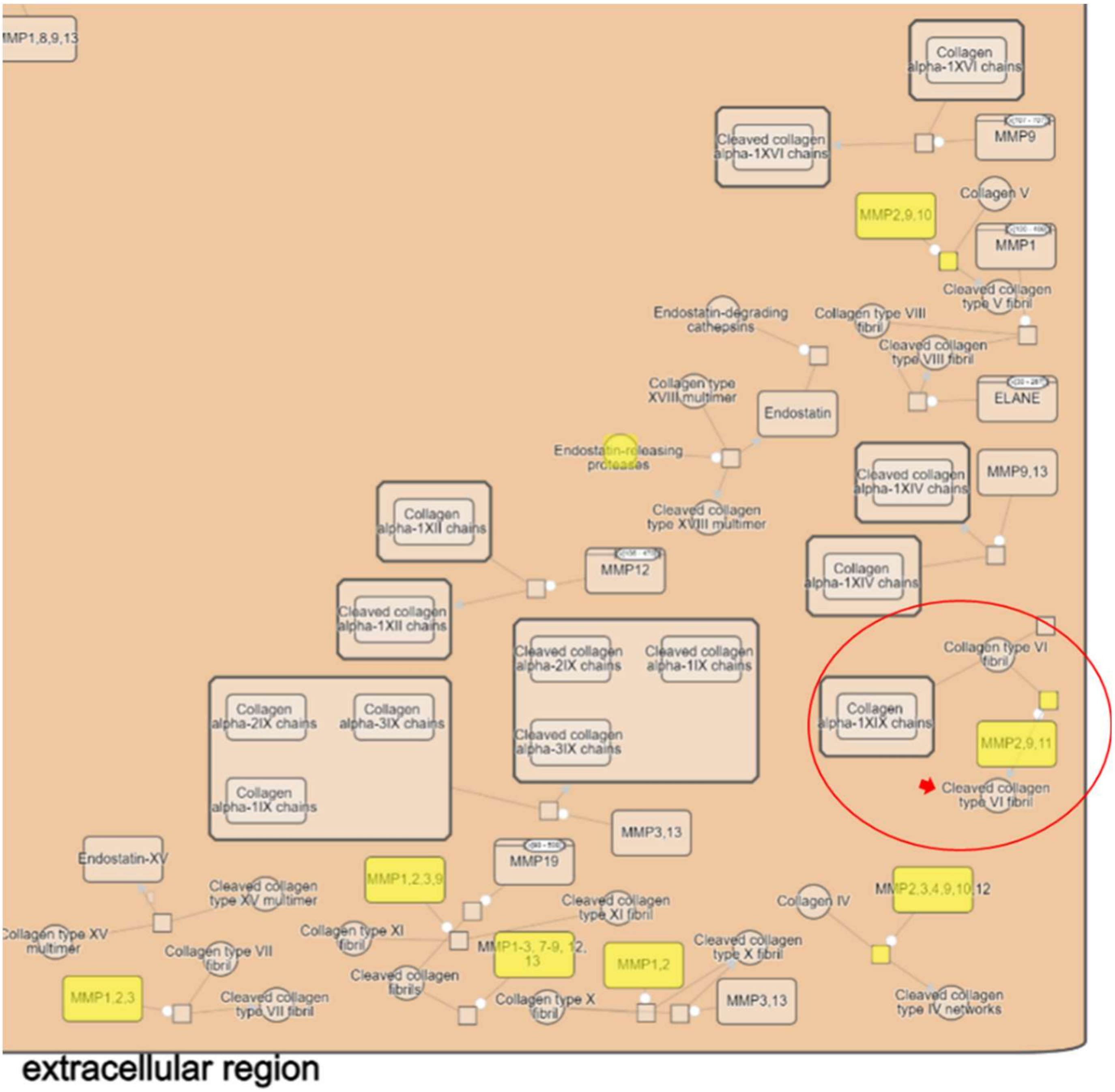

Figure S5. Biological linkage between COL6A2 and MMP2, as depicted by Pathway Commons. The portion highlighted within a red circle is regenerated in Figure 6.

symbol	GENENAME	baseMean	log2FoldChange	pvalue	imm_class
COL6A2	collagen type VI alpha 2 chain	13651.45909	-0.831	0.021	yes
CNOT1	CCR4-NOT transcription complex subunit 1	9284.111408	0.857	0.0271	no
BNC2	basonuclin zinc finger protein 2	3444.643576	-1.258	0.0219	no
ANTXR1	ANTXR cell adhesion molecule 1	3056.856683	-0.765	0.016	yes
CGN	cingulin	917.7759052	2.266	0.042	no

Table S5. Top 5 EMT-related genes. The p-value and log fold change (LFC) are the results of comparisons between HTB and LTB.

GO-term	description	count in network	strength	false discovery rate
GO:1903225	Negative regulation of endodermal cell differentiation	2 of 3	2.54	0.0125
GO:0001957	Intramembranous ossification	2 of 7	2.17	0.0348
GO:0060346	Bone trabecula formation	2 of 8	2.11	0.0380
GO:0055093	Response to hyperoxia	3 of 21	1.87	0.0052
GO:0030199	Collagen fibril organization	6 of 59	1.72	8.03e-06

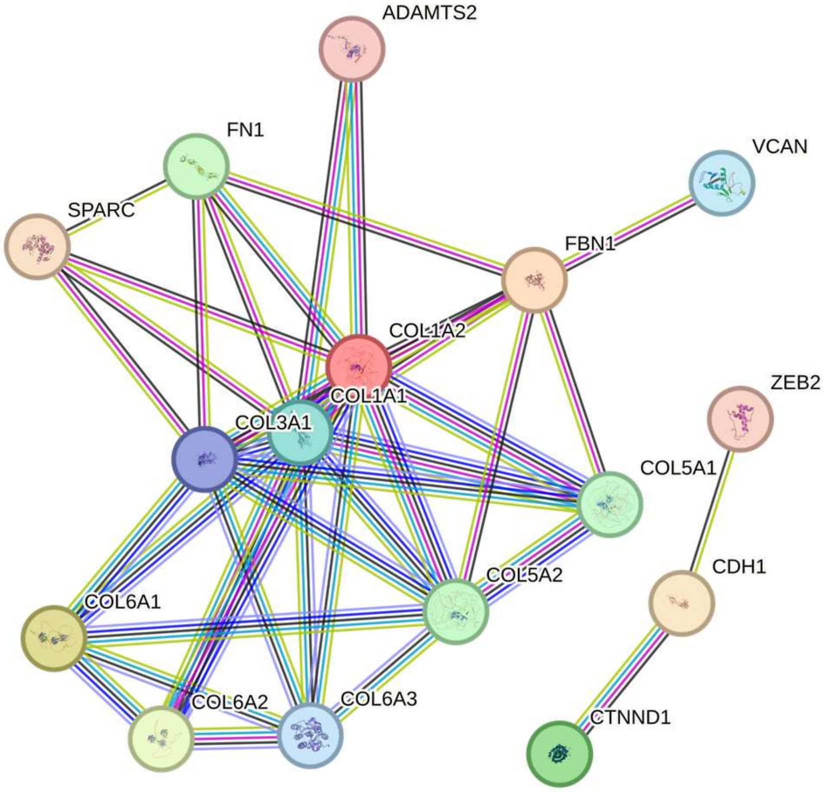

(1) hc.complete

Figure S6. STRING pathway analysis and biological process of 7 additional clustering algorithms. For Mclust is in main manuscript,

(1) hierarchical clustering with complete method,
(2) hierarchical clustering with Ward.D method
(3) k-means ,
(4) DBSCAN,
(5) KNN-Louvain community detection,
(6) hierarchical k-mean and
(7) FANNY clustering

	Biological Process (Gene Ontology)			
GO-term	description	count in network	strength	false discovery rate
O:1903225	Negative regulation of endodermal cell differentiation	2 of 3	2.64	0.0084
O:0001957	Intramembranous ossification	2 of 7	2.27	0.0261
O:0060346	Bone trabecula formation	2 of 8	2.22	0.0285
O:0032964	Collagen biosynthetic process	2 of 10	2.12	0.0386
O:0043589	Skin morphogenesis	2 of 11	2.08	0.0444

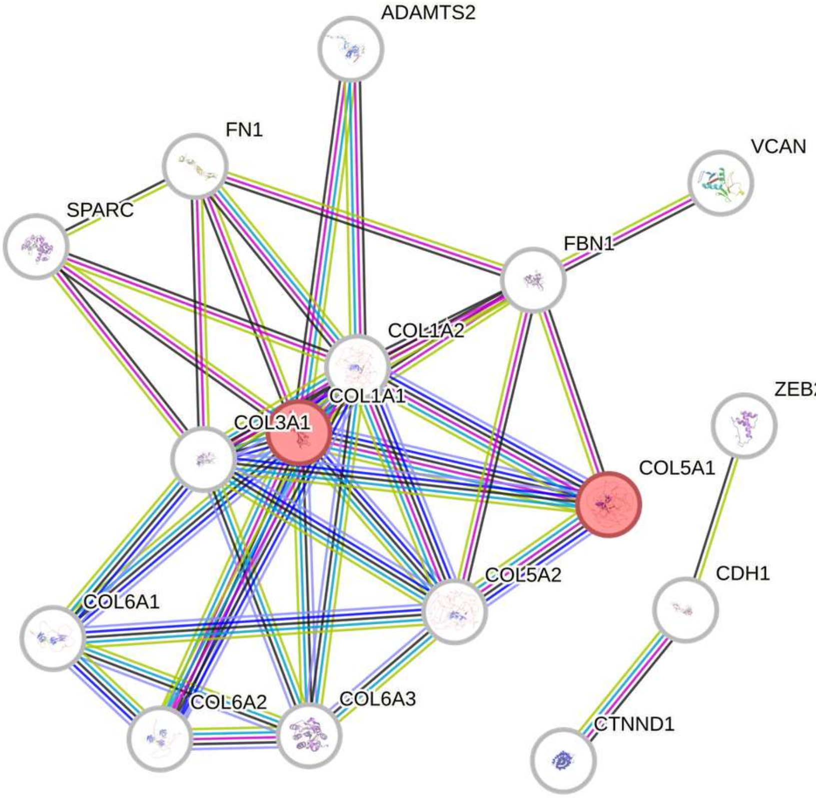

(2) hc.ward

	Biological Process (Gene Ontology)			
GO-term	description	count in network	strength	false discovery rate
GO:1903225	Negative regulation of endodermal cell differentiation	2 of 3	2.61	0.0095
GO:0001957	Intramembranous ossification	2 of 7	2.25	0.0280
GO:0060346	Bone trabecula formation	2 of 8	2.19	0.0313
GO:0032964	Collagen biosynthetic process	2 of 10	2.09	0.0408
GO:1903054	Negative regulation of extracellular matrix organization	2 of 11	2.05	0.0467

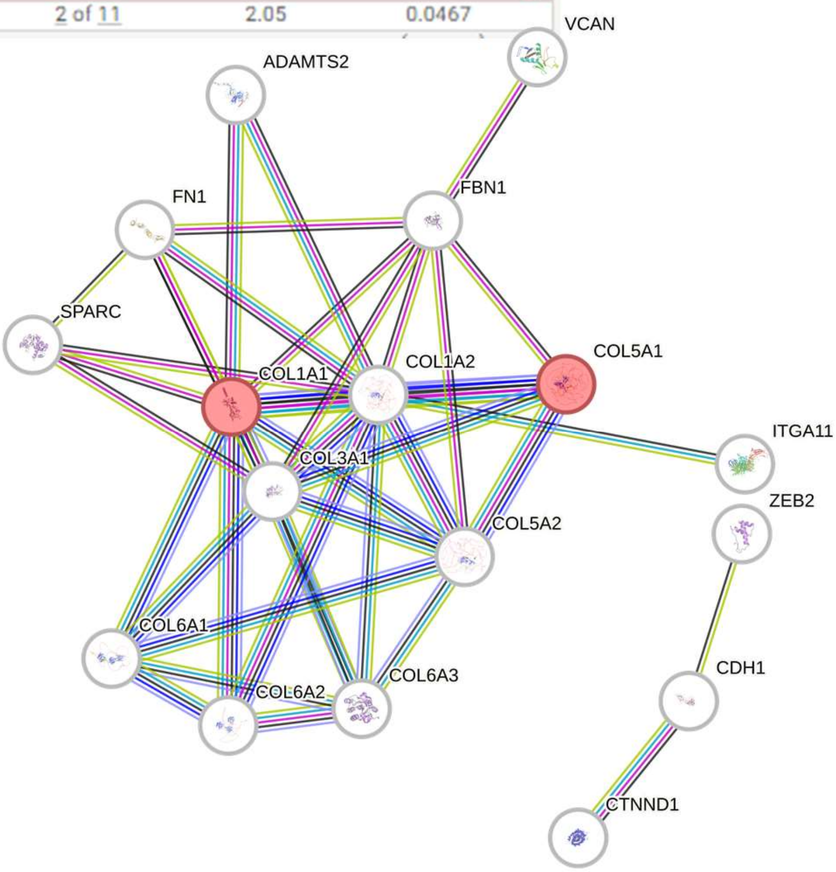

(3) Kmeans

Biological Process (Gene Ontology)				
GO-term	description	count in network	strength	false discovery rate
GO:1903225	Negative regulation of endodermal cell differentiation	2 of 3	2.27	0.0271
GO:0032964	Collagen biosynthetic process	3 of 10	1.93	0.0040
GO:0055093	Response to hyperoxia	3 of 21	1.6	0.0188
GO:0030199	Collagen fibril organization	8 of 59	1.58	2.02e-07
GO:0035987	Endodermal cell differentiation	5 of 44	1.5	0.00047

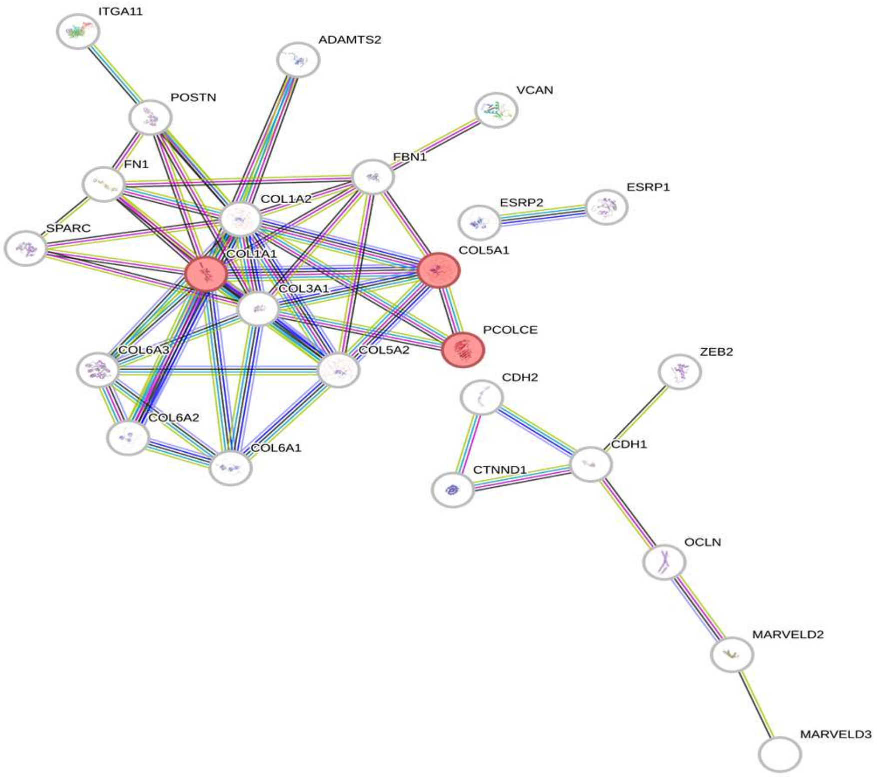

(4) DBSCAN

Biological Process (Gene Ontology)				
GO-term	description	count in network	strength	false discovery rate
GO:1903225	Negative regulation of endodermal cell differentiation	2 of 3	2.53	0.0124
GO:0001957	Intramembranous ossification	2 of 7	2.16	0.0358
GO:0060346	Bone trabecula formation	2 of 8	2.1	0.0403
GO:0032964	Collagen biosynthetic process	2 of 10	2.0	0.0499
GO:0055093	Response to hyperoxia	3 of 21	1.86	0.0058

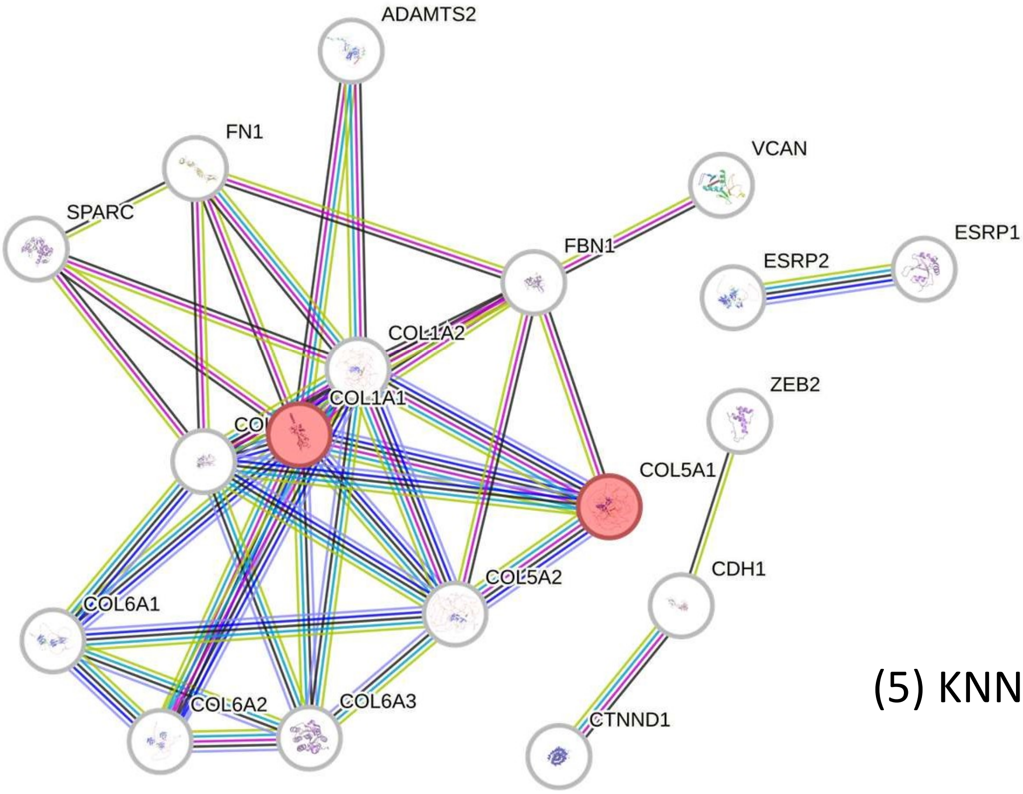

(5) KNN-Louvain

GO-term	description	count in network	strength	false discovery rate
GO:1903225	Negative regulation of endodermal cell differentiation	2 of 3	2.53	0.0124
GO:0001957	Intramembranous ossification	2 of 7	2.16	0.0338
GO:0060346	Bone trabecula formation	2 of 8	2.1	0.0403
GO:0032964	Collagen biosynthetic process	2 of 10	2.0	0.0499
GO:0055093	Response to hyperoxia	3 of 21	1.86	0.0058

Biological Process (Gene Ontology)

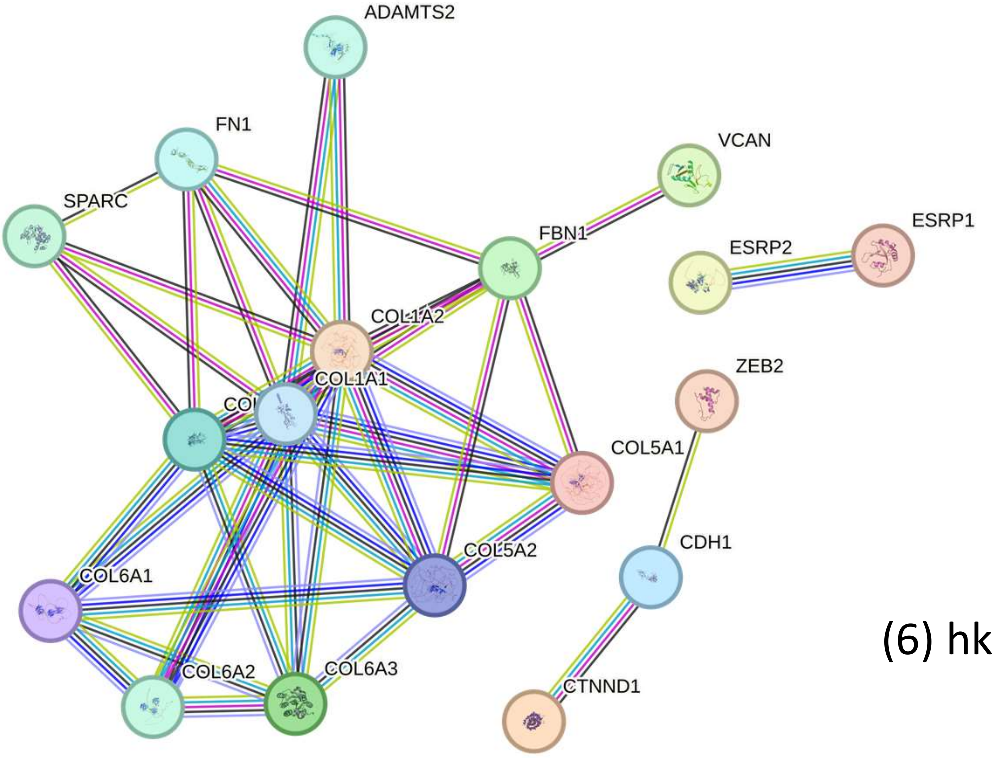

(6) hkmean

Biological Process (Gene Ontology)

GO-term	description	count in network	strength	false discovery rate
GO:1903225	Negative regulation of endodermal cell differentiation	2 of 3	2.53	0.0124
GO:0001957	Intramembranous ossification	2 of 7	2.16	0.0338
GO:0060346	Bone trabecula formation	2 of 8	2.1	0.0403
GO:0032964	Collagen biosynthetic process	2 of 10	2.0	0.0499
GO:0055093	Response to hyperoxia	3 of 21	1.86	0.0058

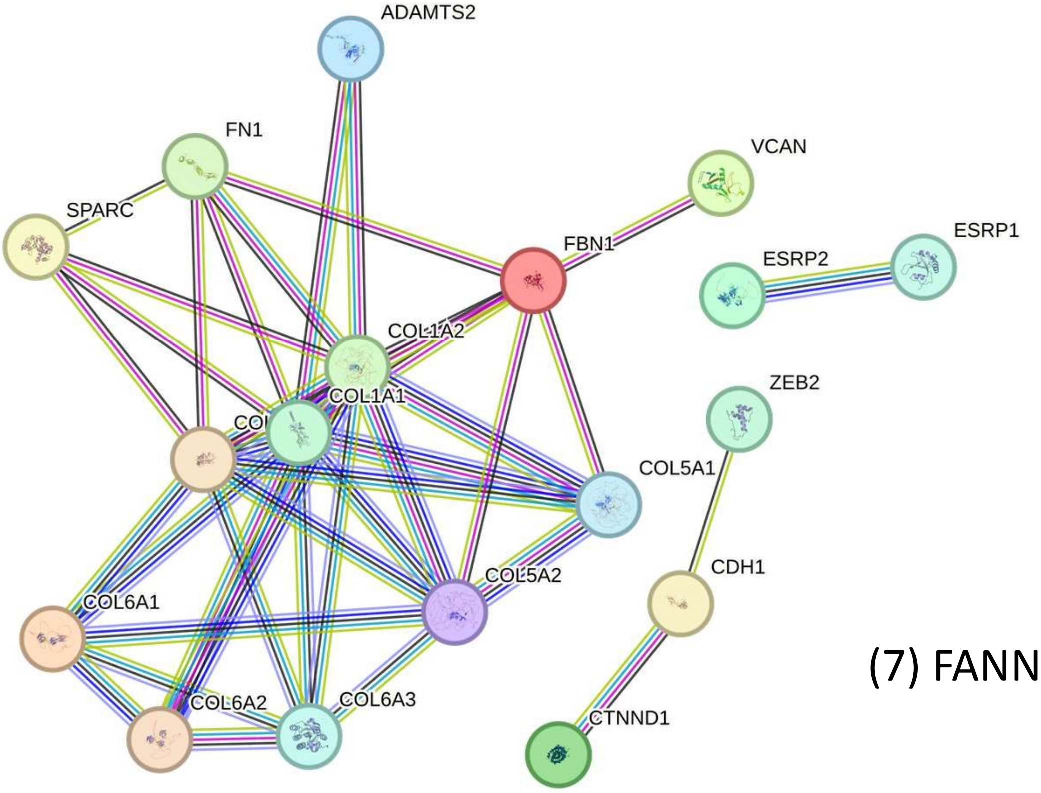

(7) FANNY

Clustering Algorithms	Total number of clusters(k)	Specific cluster	EMT-related genes (n)	Package	function
**(2) hierarchical clustering with complete method**	9	1	38	mclust	Mclust()
**(3) hierarchical clustering with Ward.D method**	9	1	30	stats	hclust()
**(4) k-means**	9	8	32	stats	hclust()
**(5) DBSCAN**	9	1	70	stats	Kmeans()
**(6) KNN-Louvain community detection**	9	1	39	fpc	dbscan()
**(7) hierarchical k-mean**	9	1	32	FNN igraph	get.knn() cluster_Louvain()
**(8) FANNY clustering**	9	1	41	factoextra	hkmeans()

Table S6. The results of the additional seven clustering algorithms. The fourth column indicates the number of EMT-related genes in a specific cluster among nine clusters. The last two columns list the R packages and functions used for each algorithm.
